# Contribution of plant-based dairy and fish alternatives to iodine nutrition in the Swiss diet: a Swiss Market Survey

**DOI:** 10.1007/s00394-024-03339-5

**Published:** 2024-03-07

**Authors:** Zulekha Abbas Khalil, Isabelle Herter-Aeberli

**Affiliations:** Institute of Food, Nutrition and Health, Laboratory of Nutrition and Metabolic Epigenetics, ETH Zürich, 8092 Zurich, Switzerland

**Keywords:** Iodine, Dairy, Fish, Plant-based alternatives, Vegan, Fortified, Market survey

## Abstract

**Purpose:**

With dairy products and fish being major sources of iodine in Switzerland, the growing popularity of plant-based alternatives may impact iodine nutrition. This study aimed to assess the iodine content in plant-based dairy and fish alternatives available in the Swiss market and compare them with conventional products.

**Methods:**

In 2022, a market survey was conducted in Zurich, Switzerland, to identify the plant-based dairy and fish alternatives available and assess their iodine content. To evaluate the impact of plant-based alternatives on iodine consumption in Switzerland, we modeled dietary scenarios by substituting the intake of dairy and fish items with plant-based alternatives. In addition, we investigated fortification with calcium, vitamins B2, B12, and D.

**Results:**

Out of 477 identified products, only four milk-alternative products were iodine fortified (median iodine concentration: 22.5 μg/100 ml). The median iodine concentration in unfortified plant-based alternatives was negligible compared to conventional dairy and fish products (milk: 0.21 vs 9.5 μg/100 ml; yogurt 0.36 vs 6.1 μg/100 g; cheese: 0.10 vs 20 μg/100 g; fish 0.50 vs 44 μg/100 g). Three portions of dairy per day as recommended by the Swiss Food Pyramid provide 25% of the RDA for iodine (150 μg/day), whereas substituting those with unfortified alternatives provides only 0.7% of the RDA.

**Conclusion:**

Only four out of 477 plant-based alternative products are iodine fortified in the Swiss market. Thus, the risk for consumers to miss out on the ca. 25% of the RDA for iodine by consuming plant-based alternatives is high, placing them at a risk for inadequate iodine intake.

**Supplementary Information:**

The online version contains supplementary material available at 10.1007/s00394-024-03339-5.

## Introduction

Iodine plays a crucial role as a micronutrient essential for growth and development [1]. It serves as a fundamental element in thyroid hormones, and iodine deficiency (ID) poses a public health risk, resulting in conditions such as goiter, hypothyroidism which increases the risk of coronary heart disease, brain damage, impaired cognitive development, and cretinism [1–4]. Consequently, impact of ID extends across all population groups and life stages. Globally, in 2020, 21 countries remained iodine deficient based on the Iodine Global Network (IGN) scorecard [5]. According to a recently published study using post-harmonization of urinary iodine concentrations, several European countries showed suboptimal iodine supply, especially in adults (7 out of 13 studies) and in pregnant women (7 out of 11 studies) [6].

Due to glaciation, the soil in Switzerland, similar to many other countries, has very low iodine content. Hence, severe goiter and cretinism were highly prevalent in Switzerland until the introduction of iodized table salt in 1922 [1]. The WHO recommended level for salt iodization is 15 to 40 ppm, and in Switzerland, since 2014, it is 25 ppm [7, 8]. However, like in many other European countries, the use of iodized salt in households and by the food industry is voluntary [1]. Most salt consumption is from processed foods, and the coverage of iodized salt in industrially processed foods is limited. While 98% of salt sold to households in Switzerland is iodized, 39% of the total salt sale is non-iodized [8].

Apart from iodized salt, dairy products, eggs, and marine fish are major dietary sources of iodine [1]. Milk and dairy products account for 30–40% of total iodine intake in the Swiss population, with conventional raw cow milk containing around 9 μg iodine/100 ml [4, 9]. Milk and dairy product consumption has been on a decline in Switzerland, as many are resorting to various forms of plant-based diets for various reasons [10]. Currently, plant-based substitutes account for a market share of 3.3% (CHF 119 million) of total dairy sales (including yogurt and cheese) and have a share of 17% of Swiss milk sales [11] Plant-based dairy alternatives are made from a variety of plant sources ranging from commonly available soy, oats, almonds, cashews, rice, and coconuts to the less common ones like lupine, chickpea, hemp, and hazelnut.

Cow milk is an important source of iodine and other nutrients specifically for pregnant and lactating women, as well as children [3, 12, 13]. Various studies have examined the nutritional composition of both milk and plant-based alternatives, considering elements like proteins, fats, calcium, vitamin D, among others. While some laboratory analyses and market surveys in different countries have assessed the iodine content of milk and its alternatives [2, 3, 14, 15], there is a need for a comprehensive market survey to assess the situation in Switzerland. Therefore, our study aimed to gather information on the iodine content of plant-based dairy and fish alternatives (both fortified and non-fortified) available on the Swiss market, and to compare their iodine content with conventional dairy and fish products. We also modeled the impact of consuming these alternatives on the iodine intake of the Swiss adult population (> 18 years old) [16] based on daily consumption of dairy products and fish and based on recommendations of the Swiss Food Pyramid (Swiss food-based dietary guidelines) for dairy and fish intake [17]. The study also surveyed the content of four other essential micronutrients (calcium, vitamin B2, vitamin B12, and vitamin D) provided by the fortified alternatives in order to compare their fortification rate with that of iodine and shed some light on the overall situation of important micronutrients provided by dairy products (naturally or, as for vitamin D, through fortification), but potentially lacking from plant-based alternatives.

## Methods

### Data collection and extraction

A cross-sectional survey of grocery retailers across Zurich, Switzerland was conducted between May 2022 and August 2022 to collect the data for available plant-based milk-alternative drinks, yogurt-, cheese-, and fish-alternative products and to investigate the extent of iodine fortification as well as the fortification with calcium and vitamins B2, B12, and D. The products included were those marketed as alternatives for milk, dairy products, and fish. The survey included eight retail supermarket chains: Coop [18], Migros [19], Alnatura [20], Lidl [21], Aldi [22], Spar [23], Volg [24], and Denner [25]; one health food store, Coop Vitality [26]; three online grocery stores: Farmy [27], Hello Vegan [28], and Mr. Vegan [29]; and one local vegan food store: Eva’s Apples [30]. The retail chains Coop and Migros have a combined market share of around 70% in Switzerland. Denner has a market share of 9.2%, Volg has a share of 4.2%, whereas other chains such as Lidl and Aldi occupy 2.8% of the Swiss market share [31, 32].

Initially, data were collected by visiting one major branch of each retail chain in Zurich, Switzerland. After that, we checked the online websites of all retailers to validate the nutritional information and ensure the completeness of products. The online search of products was carried out on the respective retailer websites by clicking on “Menu” and then searching for categories labeled either “vegan” or “plant-based” or “dairy-free.” If the website did not have a categorized menu then the search bar was used for words such as “vegan,” “plant-based,” “milk-alternative,” “almond,” “soya,” “cashew,” “coconut,” “rice,” “pea,” “vegan fish,” and “plant-based fish.” We surveyed the following alternative products for our study: milk-alternative drinks, yogurt alternatives, cheese alternatives, and fish alternatives. Products from both long-life and refrigerated sections were included in this study. Furthermore, organic products as well as non-organic products were included. However, flavored milk-alternative drinks, products for infants (age 0–2 years), products that are not dairy alternatives (tinned coconut milk, coconut water), and products that are sold as part of a meal/meal replacement were excluded from this study.

Smartphone cameras were used to take pictures of in-store products and to record on-pack nutrition information. All the product information and nutritional information from the photos and from the online websites were transferred into a Microsoft Excel Spreadsheet (Redmond, WA, USA: Microsoft Corp.) [33]. The data extraction for each product category (milk, yogurt, cheese, and fish alternatives) was done separately. The data recorded included product name, brand, product type, organic/non-organic, sweetened/unsweetened, net weight, and micronutrient data from the nutrition label. The products were checked for the addition of micronutrients to the product as indicated on the nutrition label, including calcium, iodine, vitamin B2, vitamin B12, and vitamin D. Milk-alternative drinks were categorized based on the main characterizing plant ingredient used and the product type for each item was recorded (Nature, Barista, with Hazelnut, Original, etc.). Similarly, yogurt-, cheese-, and fish-alternative products were also categorized based on the main characterizing plant ingredient used.

### Iodine content and content of other micronutrients

#### Fortified alternatives

The median iodine concentration of fortified products was calculated from the declared iodine concentration on all fortified products. Likewise, the median concentrations of other micronutrients such as calcium, vitamin B2, vitamin B12, and vitamin D were calculated based on the micronutrient content provided on the nutrition labels of fortified products.

#### Unfortified alternatives

Concentration of iodine from unfortified products was calculated factorially. The Swiss Food Composition Database was used to derive the iodine content in 100 g of the characterizing plant ingredient [34]. According to the Swiss food labeling guidelines, the characterizing ingredient (e.g., oat flakes) in products is declared as a percentage on the final product label [35]. This percentage along with the iodine content in 100 g of characterizing ingredient was then used to calculate the iodine content in 100 g or 100 ml of product. For example, from the Swiss Food Composition Database, it was noted that 100 g of oat flakes contains 0.5 µg of iodine. A product containing 10% oats was considered equivalent to 10 g of oats per 100 ml of product. Hence, the iodine content in 100 ml of the product will be computed to be 0.05 µg per 100 ml of product.

#### Animal-based products

Concentration of iodine and other micronutrients in animal-based products was obtained from the Swiss Food Composition Database for cow’s milk, yogurt, cheese, and fish [34]. Median values for iodine and the other four micronutrients were computed for each category of animal-based products.

### Dietary scenarios

In order to understand the effect of the replacement of dairy and fish with vegan alternatives on iodine intake, we modeled three dietary scenarios where we replaced the intake of dairy and fish products in the Swiss population with plant-based alternatives. First, we calculated the iodine intake in the Swiss population (adults > 18 years old) based on the daily dairy and fish intake. A nationally representative survey conducted in 2014–15, the National Nutrition Survey menuCH, provides the data on intake of milk, yogurt, cheese, and fish among the Swiss adult population [16]. These intake data were then used to calculate the iodine intake per day from animal-based products using the nutritional composition in the Swiss Food Composition Database as described above [34]. The amounts calculated this way were used as reference scenarios REF-dairy-obs and REF-fish-obs. Similarly, we calculated iodine intakes if the recommendations of the Swiss Food Pyramid [17] were followed, i.e., one portion of milk (200 g), one portion of yogurt (175 g), and one portion of cheese (45 g) per day as well as two portions of 110 g fish per week, which equals 31.4 g fish per day. The achieved amounts were used as reference scenarios REF-dairy-rec and REF-fish-rec.

For both options, we then modeled dairy intake based on the following scenarios: (1) replacing the daily milk intake (based on actual intake or recommendation) with a fortified milk alternative and the amounts of yogurt and cheese with unfortified alternatives, and (2) replacing the amount of all three dairy products with an equivalent amount of unfortified alternatives. For fish intake only one scenario was possible for each of the two options above, namely, replacing the daily fish intake or recommendation by an unfortified alternative.

After the dietary modeling, we calculated the percentage of iodine obtained per day from the modeled intake based on the Recommended Dietary Allowance (RDA) of iodine for adults (18 years old) of 150 μg/day by the World Health Organization (WHO) [36].

### Statistical analysis

All data for conventional dairy and fish products and plant-based alternatives were statistically analyzed using IBM SPSS Statistics for Windows Version 28.0 (Armonk, NY, USA: IBM Corp.) with the significance level set at *P* < 0.05 [37]. The normality of data distribution was verified through the Shapiro–Wilk test as well as visual inspection of histogram plots. As the data were not normally distributed, we report the median (and 25th, 75th percentile, where possible). The non-parametric Mann–Whitney *U* test for independent samples was performed to compare the following: (i) micronutrient concentration (iodine, calcium, vitamin B2, vitamin B12, and vitamin D) in fortified dairy and fish alternatives with conventional dairy and fish, (ii) median iodine concentration of unfortified alternatives with conventional dairy and fish, and (iii) median iodine concentration of unfortified milk alternatives with iodine-fortified milk alternatives. Data collection, graphs, and calculations were performed on Microsoft Excel (Redmond, WA, USA: Microsoft Corp.) [33].

## Results

After finalizing the total list of products, we categorized each product type based on their characterizing plant ingredient, e.g., oats based, almond based, soy based, rice based, etc. (online resource: Supplementary Table S1a–d). A total of 477 products were identified for the study, of which there were 170 milk-alternative drinks, 113 yogurt alternatives (both flavored and unflavored), 166 cheese alternatives, and 28 fish alternatives. An overview of the distribution of iodine in milk-alternative products based on their characterizing ingredient is given in Table 1. Furthermore, a consolidated list of all products included is provided in the online resource (Supplementary Table S1a–d).

**Table 1:** Number of products and median concentration of iodine in iodine-fortified plant-based milk (per 100 ml)

Alternatives products	Total number of products	Number of products fortified with iodine	Percentage fortified with iodine (%)	Iodine concentration of fortified products (µg/100 ml)		
				Median	Range	25th, 75th Percentile^a^
Milk						
Oat	51	4	7.84	22.50	22.50	22.50, 22.50
Almond	19	0	0.00	–	–	–
Soy	23	0	0.00	–	–	–
Rice	28	0	0.00	–	–	–
Coconut	12	0	0.00	–	–	–
Cashew	6	0	0.00	–	–	–
Pea	7	0	0.00	–	–	–
Others	24	0	0.00	–	–	–
Total products	170	4	2.35	–	–	–
Median				22.50	22.50	22.50, 22.50

^a^Test for normality using the Shapiro–Wilk test in IBM SPSS [37]

It was observed that 76% of milk alternatives were organic (*n* = 129) and 24% had added sugar (*n* = 40). Of the yogurt alternatives, 58% (*n* = 65) were identified to be organic, 71% (*n* = 80) were flavored alternatives, and 67% (*n* = 76) had added sugar. In the case of cheese alternatives, of the total of 166 products, 46% (*n* = 77) were identified to be organic. The most widely available replacements were cheese slices (*n* = 33), followed by cheese spread (*n* = 33) and hard/semi-hard cheese blocks (*n* = 29). Only 29% (*n* = 8) of fish alternatives were of organic origin. The most widely available replacements out of the 28 fish-alternative products reported were tuna (*n* = 10) and salmon (*n* = 7).

### Micronutrient concentrations in fortified products

Table 2 specifies the median and range of concentrations of iodine, calcium, and vitamins B2, B12, and D in fortified plant-based milk, yogurt, cheese, and fish-alternative products in comparison with conventional dairy and fish products. The median iodine content in unfortified plant-based dairy and fish alternatives in comparison with animal products is presented in Table 3. The data extracted for animal-based products from the Swiss Food Composition Database are provided in the online resource (Supplementary Table S2a–d).

**Table 2:** Median concentration of all five micronutrients—iodine, calcium, Vitamin B2, Vitamin B12, and Vitamin D in dairy and fish products and plant-based fortified alternatives

Category	Total products	Number fortified	Iodine (µg/100 ml or 100 g)		Number fortified	Calcium (mg/100 ml or 100 g)		Number fortified	Vit B2 (mg/100 ml or 100 g)		Number fortified	Vit B12 (μg/100 ml or 100 g)		Number fortified	Vit D (μg/100 ml or 100 g)	
			Median (range)	*P*-value*		Median (range)	*P*-value*		Median (range)	*P*-value*		Median (Range)	*P*-Value*		Media (range)	*P*-Value*
Milk																
Cow’s milk^a^	NA	NA	9.5 (3.1–10)	0.006	NA	120 (120–130)	0.069	NA	0.2 (0.16–0.22)	0.056	NA	0.21 (0.10–0.33)	< 0.001	NA	0.1 (0.00–0.10)	< 0.001
Milk alternatives	170	4	22.50 (22.5)		32	120 (60–120)		13	0.21 (0.1–0.21)		18	0.38 (0.20–0.40)		18	0.76 (0.40- 1.10)	
Yogurt																
Cow’s milk yogurt^a^	NA	NA	6.1 (5.5–21)	NA	NA	120 (100–160)	0.493	NA	0.16 (0.12–0.34)	0.015	NA	0.37 (0.15–0.50)	0.792	NA	0.1 (0.00–0.20)	< 0.001
Yogurt alternatives	113	0	NA (NA)		20	120 (96–120)		12	0.21 (0.21)		17	0.38 (0.30–0.38)		17	0.75 (0.60–0.75)	
Cheese																
Cow’s milk cheese^a^	NA	NA	20 (0.00–40)	NA	NA	550 (68–1340)	0.003	NA	0.35 (0.00–0.81)	NA	NA	1.5 (0.00–2.18)	< 0.001	NA	0.5 (0.00–0.90)	NA
Cheese alternatives	166	0	NA (NA)		5	200 (54–260)		0	NA (NA)		24	1.9 (1.30–2.50)		0	NA (NA)	
Fish																
Fish products^a^	NA	NA	44 (0.00–180)	NA	NA	23 (8–400)	NA	NA	0.08 (0.04–0.25)	0.091	NA	2 (0.00–20.9)	0.059	NA	2.3 (0.00–22.1)	0.55
Fish alternatives	28	0	NA (NA)		0	NA (NA)		1	0.4 (0.4)		3	0.8 (0.71–1.25)		1	1.5 (1.5)	

*NA* Not applicable

*P-value calculated from Mann–Whitney non-parametric test for two independent samples for comparison of iodine content in animal-based dairy or fish products with that of fortified plant alternatives [37]

^a^Median values of micronutrients obtained from Swiss Food Composition Database for various cow milk and fish products [34]

**Table 3:** Median iodine concentration of unfortified alternatives in comparison to dairy and fish

Category	Median iodine concentration (μg/100 ml or 100 g)	25th, 75th Percentile^b^	*P*-value*
Milk			
Cow milk	9.50^a^	9.20, 10.00	0.001
Unfortified alternatives	0.211	0.074, 0.38	
Yogurt			
Cow milk yogurt	6.10^a^	6.00, 8.50	< 0.001
Unfortified alternatives	0.36	0.04, 0.71	
Cheese			
Cow milk cheese	20.00^a^	10.50, 35.00	< 0.001
Unfortified alternatives	0.105	0.047, 2.04	
Fish			
Fish products	44.00^a^	14.00, 85.00	< 0.001
Unfortified alternatives	0.57	0.27, 2.39	

*P-value calculated from Mann–Whitney *U* non-parametric test for two independent samples for comparison of iodine content in dairy or fish products with that of unfortified plant alternatives [37]

^a^Median values of iodine obtained from Swiss Food Composition Database for various cow milk and fish products [34]

^b^Test for normality using the Shapiro–Wilk test in IBM SPSS [37]

The median iodine concentration of each plant type of unfortified plant-based alternative is presented in the online resource (Supplementary Table S3). The iodine content in 100 g of characterizing ingredients extracted from the Swiss Food Composition Database is also provided in the online resource (Supplementary Table S4).

#### Milk alternatives

In total, 19% (*n* = 32) of all the milk alternatives were fortified with at least one micronutrient. Calcium was found to be the most commonly used fortificant, as all of the 32 fortified milk-alternative products were calcium fortified as opposed to iodine (*n* = 4), vitamin B2 (*n* = 13), vitamin B12 (*n* = 18), and vitamin D (*n* = 18).

There was only one brand (4 products) of oat-based, iodine-fortified milk alternatives which all contained 22.5 μg iodine/100 ml. Thus, the median iodine concentration in fortified milk-alternative products was significantly higher than that in Swiss cow milk (9.5 μg/100 ml, *P* = 0.006) (compare Table 2). While calcium and vitamin B2 fortification levels in milk-alternative products were comparable to the respective values in cow milk, the added concentrations of vitamin B12 and vitamin D surpassed the natural concentration in cow milk (compare Table 2).

#### Yogurt alternatives

No iodine-fortified yogurt-alternative product was found in the market. We identified 20 yogurt-alternative products fortified with calcium, 12 with vitamin B2, 17 with vitamin B12, and 17 with vitamin D. Fortification levels were found to be close to the natural concentration of cow milk products for calcium and vitamin B12 but were significantly higher for vitamin B2 and vitamin D (compare Table 2).

#### Cheese alternatives

Similar to yogurt alternatives, no plant-based cheese alternatives were fortified with iodine. Our survey found only 5 cheese-alternative products fortified with calcium and 24 with vitamin B12. While the calcium concentration of the alternative products was lower, the vitamin B12 concentration was comparable to that of cow milk cheese (compare Table 2).

#### Fish alternatives

No plant-based alternatives of fish were fortified with iodine. We identified 1 fish-alternative product fortified with vitamin B2, 3 with vitamin B12, and 1 with vitamin D. While the concentration of vitamin B2 was higher in the alternative product, vitamin B12 and vitamin D concentrations were lower, even though none of those results were significant due to the low numbers (compare Table 2).

### Iodine concentration in unfortified alternatives

The iodine content in the 473 unfortified alternatives identified in the Swiss market was calculated factorially on the basis of the percentage of characterizing ingredients present in the products. We observed that the median iodine concentration of unfortified milk alternatives was significantly lower in comparison to cow milk (*P* = 0.001) (Table 3) and fortified milk alternatives (*P* = 0.006). Similarly, the median iodine concentration of unfortified yogurt, cheese, and fish alternatives was low in comparison to cow milk yogurt and cheese, as well as fish (all *P* > 0.05) (Table 3).

### Iodine intake and modeling based on Swiss adult intake of dairy and fish products and recommendations by the Swiss Food Pyramid

#### Iodine intake based on Swiss adult intake (Table 4)

**Table 4:** Iodine intake in the Swiss adult population (adults > 18 years) based on the per day dairy and fish observed intake

Category	Product	Swiss daily intake (ml/day or g/day)^a^	Iodine content (µg/100 ml or 100 g)	Iodine per day based on intake (µg/day)
Milk	Cow’s milk	119.40	9.5^b^	11.34
	Fortified milk alternative		22.5^c^	26.86
	Unfortified milk alternative		0.21^d^	0.25
Yogurt	Yogurt from cow’s milk	74.60	6.1^b^	4.55
	Fortified yogurt alternative		NA	NA
	Unfortified yogurt alternative		0.36^d^	0.27
Cheese	Cheese from cow’s milk	63.60	20.00^b^	12.72
	Fortified cheese alternative		NA	NA
	Unfortified cheese alternative		0.10^d^	0.06
Fish	Fish	35.80	44.00^b^	15.75
	Fortified fish alternative		NA	NA
	Unfortified fish alternative		0.57^d^	0.20

^a^Swiss adult intake data obtained from the National Nutrition Survey menuCH of 2014–15 [16]

^b^Median values of iodine obtained from Swiss Food Composition Database for various cow’s milk and fish products [34]

^c^Median values of iodine obtained from the nutrition label of iodine-fortified plant-alternative products

^d^Concentration of iodine from unfortified products calculated factorially. The Swiss Food Composition Database was used to derive the iodine content in 100 g of the characterizing ingredient [34]

An average daily intake of 119.4 ml of cow milk, 74.6 g of yogurt, 63.6 g of cheese, and 35.8 g of fish was observed among the Swiss adult population. The amounts of iodine provided by those portions are reported in Table 4. Similarly, the amount of iodine provided by the same portions of fortified (only milk) and unfortified alternative products is given in Table 4.

#### Iodine intake based on Swiss Food Pyramid recommendations (Table 5)

**Table 5:** Iodine obtained from a daily recommended portion of dairy and fish products based on the Swiss Food Pyramid [17]

Category	Product	Recommended portion per day (Swiss Food Pyramid) (ml or g)^a^	Iodine content (µg/100 g)	Iodine in daily recommended portion (µg/portion per day)
Milk	Cow’s milk	200	9.50^b^	19.00
	Fortified milk alternative		22.50^c^	45.00
	Unfortified milk alternative		0.21^d^	0.42
Yogurt	Yogurt from cow’s milk	175	6.10^b^	10.67
	Fortified yogurt alternative		NA	NA
	Unfortified yogurt alternative		0.36^d^	0.63
Cheese	Cheese from cow’s milk	45	20.00^b^	9.00
	Fortified cheese alternative		NA	NA
	Unfortified cheese alternative		0.10^d^	0.04
Fish	Fish	31.42*	44.00^b^	13.82
	Fortified fish alternative		NA	NA
	Unfortified fish alternative		0.57^d^	0.18

*Recommended portion of fish in a week by the Swiss Food Pyramid, considering one consumes fish twice per week, i.e., 110 g twice per week = 220 g/week = 31.42 g/day [17]

^a^Portion of cow milk products recommended based on Swiss Food Pyramid [17]

^b^Median values of iodine obtained from the Swiss Food Composition Database for various cow’s milk and fish products [34]

^c^Median values of iodine obtained from the nutrition label of iodine-fortified plant-alternative products

^d^Concentration of iodine from unfortified products calculated factorially. The Swiss Food Composition Database was used to derive the iodine content in 100 g of the characterizing ingredient [34]

An average daily intake of 200 ml of cow milk, 175 g of yogurt, 45 g of cheese, and 31.42 g of fish is recommended by the Swiss Food Pyramid. The amounts of iodine provided by those portions are reported in Table 5. Similarly, the amount of iodine provided by the same portions of fortified milk alternative and unfortified yogurt and cheese alternatives is given in Table 5.

#### Dietary modeling (Table 6)

**Table 6:** Modeled dietary scenarios based on Swiss observed intake and Swiss Food pyramid recommendations of dairy and fish per day

Scenario No.	Scenarios	Swiss dairy or fish observed intake per day (g/day)^a^	Total iodine based on observed intake (µg/day)^b^	Percentage of iodine from Swiss intake based on WHO recommendations (% of 150 µg/day)^1^
REF-dairy-obs	Intake of dairy per day observed in Swiss population: milk + yogurt + cheese	257.6	28.61	19.07
I	One fortified milk alternative and two unfortified alternatives (yogurt and cheese)	257.6	27.20	18.13
II	Three unfortified alternatives (milk, yogurt, and cheese)	257.6	0.586	0.31
REF-fish-obs	Intake of fish per day observed in Swiss population	35.8	15.75	10.50
III	Unfortified fish alternative per day	35.8	0.20	0.13

		Swiss recommended portion per day (Food Pyramid) (g/day)^c^	Total Iodine based on recommended intake (µg/day)^d^	Percentage of iodine from recommended portions based on WHO recommendations (% of 150 µg/day)^1^
REF-dairy-rec	Recommended three portions of dairy: milk, yogurt, and cheese	420	38.67	25.78
I	One fortified milk alternative and two unfortified alternatives (yogurt and cheese)	420	45.677	30.45
II	Three unfortified alternatives (milk, yogurt, and cheese)	420	1.099	0.73
REF-fish-rec	Intake of fish per day based on recommendations	31.42*	13.82	9.22
III	Unfortified fish alternative per day	31.42*	0.18	0.12

*Recommended portion of fish in a week by the Swiss Food Pyramid, considering one consumes fish twice per week, i.e., 110 g twice/week = 220 g/week = 31.42 g/day [17]

^a^Swiss adult daily average intake data obtained from the National Nutrition Survey menuCH of 2014–15–119.5 ml/day of milk + 74.6 g/day of yogurt + 63.6 g/day of cheese = 257.6 g; and 35.8 g/day of fish [16]

^b^Iodine obtained from the daily dairy and fish intake in the Swiss adult population (adults > 18 years old), ref Table 4

^c^Recommended portion of dairy per day according to the Swiss Food Pyramid–200 ml/day of milk + 175 g/day yogurt + 45 g/day of cheese = 420 g; and 31.42 g/day of fish [17]

^d^Iodine obtained from the recommended dairy and fish portion (adults > 18 years), ref Table 5

^1^Recommended Dietary Allowance (RDA) for Iodine is 150 μg/day recommended by WHO [36]

We modeled iodine intake when replacing certain portions of dairy and fish intake by plant-based alternatives (fortified or unfortified) (compare Table 6). We showed that total iodine intake remained similar when replacing the daily portion of cow milk by one of the four fortified alternatives and yogurt and cheese by unfortified alternatives. When replacing all dairy products by unfortified alternatives, however, total iodine intake covers only 0.31% of the WHO recommendations compared to 19.07% in the dairy scenario. Similarly, unfortified fish-alternative products provide only 0.13% of the WHO recommendations compared to 10.50% by fish. A similar picture is seen when using Swiss Food Pyramid intake recommendations instead of intake as the basis of the calculation (compare Table 6).

## Discussion

This is the first study investigating iodine content in plant-based dairy and fish-alternative products as compared to dairy and fish products on the Swiss market and to assess iodine intake of the population based on those products.

### Iodine in dairy and fish products

Apart from iodized salt, dairy products are one of the major sources of iodine for the Swiss population. It can contribute 30%–40% of the total iodine intake [9]. Even though marine fish is also a good source of iodine in general, due to the limited consumption in Switzerland its contribution to the total iodine intake is limited (ca. 6%) [38]. Based on our results, if Swiss adults consume dairy and fish as recommended by the Swiss Food Pyramid, that is, three portions of dairy per day and two portions of fish per week, they would reach approximately 36% of the RDA for iodine of 150 μg/day. However, based on the National Nutrition Survey menuCH 2014–15 [16], the intake of dairy and fish products among Swiss adults is lower than the recommendations. Nevertheless, based on the consumed amounts of dairy and fish in Switzerland, the two food groups still contribute to almost 30% of the total RDA for iodine. According to a similar market survey conducted in the United Kingdom (UK) in 2021 [2], three portions of dairy per day (200 ml of milk, 150 g of yogurt, and 30 g of cheese) and two portions of fish per week (280 g per week) provide approximately 98% of the RDA for iodine. However, the reported concentrations of iodine in Swiss cow milk and cow milk from the UK differ considerably, with UK products having higher concentrations, which explains these discrepancies [2]. Iodine content of cow milk and dairy products is largely influenced by feeding and farming practices and regulations and thus can vary largely between countries. [39]

Marine fish are one of the major natural dietary sources of iodine [1]. The median iodine concentration of fish available in Switzerland (44 μg/100 ml) is lower in comparison to the median iodine concentration of white and oily fish in the UK (120 μg/100 ml) [2]. Freshwater fish have much lower iodine content than marine fish [1]. Since a big proportion of the fish available in Switzerland is freshwater fish, the overall median iodine concentration is reduced as is the iodine intake from fish.

### Iodine fortification in the market

Our survey shows that plant-based dairy and fish alternatives available on the Swiss market are poor sources of iodine, except for the few available iodine-fortified alternatives. Although the median iodine concentration in the four fortified milk alternatives in Switzerland is higher than the median iodine concentration of cow milk, considering the low availability, the Swiss adult population consuming milk alternatives may be at risk of inadequate iodine intake.

Besides the four milk-alternative products, no other product category, alternatives for yogurt, cheese, or fish, was fortified with iodine. In comparison, the market survey conducted in the UK, reported a higher, although still low proportion of iodine-fortified products milk alternatives (29 out of the 146 identified) [2]. However, they also identified only a small number of yogurt alternatives (*n* = 4) fortified with iodine was identified and not iodine-fortified cheese nor fish alternatives [2]. Iodine obtained from consuming unfortified yogurt, cheese, and fish alternatives, similar to milk alternatives, is negligible as compared to iodine obtained from consuming cow milk yogurt and cheese or fish.

The reported average daily consumption of dairy by Swiss adults according to menuCH is 258 g/day [16]. This provides about 19% of the RDA for iodine. By replacing the consumed amount of milk by a fortified plant-based alternative while yogurt and cheese are replaced by unfortified alternatives, a similar intake of 18% of the RDA can be achieved. However, by replacing all dairy products by unfortified plant-based alternatives, this value is reduced to 0.3% of the RDA. By following the Swiss Food Pyramid guidelines, amounting to 420 g/day of dairy [17], these amounts are increased to 25%, 30%, and 0.7% of the RDA, respectively. These calculations clearly show the potential of fortified products to cover iodine needs of the Swiss population, but at the same time, that the lack of products available on the market limits this potential.

Both based on the average daily intake and the Swiss recommendations, iodine intake from fish covers ca. 10% of the RDA for adults. However, since only 50% of the population eats fish once or more in a week [1], this does not seem to be a reliable source for the entire population. Furthermore, when fish intake is replaced by plant-based fish alternatives, this is reduced even more to around 0.13% of the RDA for iodine. Thus, by replacing both dairy and fish by plant-based alternatives, iodine intake may be reduced from almost 30% of the adult RDA to less than 1%. In order to cover the missing 45 µg/day of iodine, e.g., with iodized table salt an additional amount of ca. 2 g of salt would be required at the current fortification level of 25 mg of iodine/kg of salt.

### Availability of iodine-fortified alternatives, cost, and labeling

Our results suggest that iodine-fortified plant-based dairy and fish-alternative products can significantly contribute to an adequate iodine supply. However, currently the availability is very limited. This problem does not seem to affect only Switzerland. The lack of iodine fortification in plant-based alternatives is a pattern observed in many market surveys across the world, including the UK [3], Australia [12], Norway [15], and the USA [40]. Consumers are often not aware of the importance of iodine adequacy in daily nutrition. Lack of awareness about the importance of iodine in the diet is also one of the major reasons for the reluctance of the food industry to produce iodine-fortified products [1].

Considering the low iodine status identified in vegan populations [41, 42], strategies for improvement are essential, especially in the light of the ongoing trend toward a plant-based nutrition. All stakeholders including the food industry, government food, and health departments, and public health managers should come together and encourage consumers to choose and prefer iodine-fortified products. Simultaneously, food manufacturers should be encouraged to use iodized salt for food production in processed foods and organic products. Together, introducing a higher number of fortified plant-based alternatives and an increased awareness on the importance of iodine nutrition in the general population are needed to improve this situation.

In our survey, we observed that in many of the plant-based alternatives and even in many dairy products, salt (as table, sea, and cooking salt) was an added ingredient; however, the added salt is not iodized salt. The addition of iodized salt to those products could help increase iodine intake. On the other hand, it was observed that some of the fish alternatives in our survey contained seaweed and few dairy alternatives had *Lithothamnium calcareum*—a seaweed added as a source of calcium. However, the concentration or percentage of seaweed added was not declared on the food label and could therefore not be taken into consideration in the calculations. A recent laboratory-based analysis of 27 milk alternatives in Switzerland reported a median iodine concentration of 0.62 µg/100 ml, which is slightly higher than the median iodine concentration of iodine unfortified milk alternatives (0.21 µg/100 ml) calculated in our study [10]. However, it should be noted that the laboratory-based study in Switzerland also included milk alternatives with added *Lithothamnium calcareum* for analysis [10]. According to two laboratory-based studies, plant-based alternatives containing *Lithothamnium calcareum* had considerably higher iodine concentrations than that of other unfortified alternatives [3, 10]. Despite the discrepancy between the two results, compared to milk and dairy products the content is very low in both and the conclusions drawn would not be altered even if our results slightly underestimated iodine content in the alternative products.

### Iodine in unfortified products

We calculated Iodine in unfortified plant-based alternatives factorially using the Swiss Food Composition Database [34]. We observed that the median concentration of naturally available iodine in unfortified plant-based dairy alternatives in Switzerland is lower than reported iodine in unfortified plant-based dairy alternatives, e.g., in the UK (milk: 0.21 vs 0.56 μg/100 ml; yogurt: 0.36 vs 0.84 μg/100 g; cheese: 0.10 vs 0.80 μg/100 g) [2]. The iodine in unfortified plant-based alternatives in the UK market survey was also calculated factorially but using the UK Food Tables. Upon comparison, we noted that the iodine concentration in some of the characterizing ingredients in the Swiss Food Composition Database was lower than that in the UK Food Tables (e.g., cashew nuts kernel 5.0 μg vs 11.0 μg/100 g) [2, 43]. As cashew nuts are grown neither in the UK nor in Switzerland, it is questionable whether those differences really exist. The nutrient composition of generic products varies depending on soil, growing conditions, etc. Thus, a given value does most certainly not represent the exact content in all cashew nuts. Nevertheless, since the amount of iodine in the plant-based products is still low in both surveys, this may not significantly influence the interpretation of our results.

### Other micronutrients in plant-based alternatives

We also present the concentration of four other fortificants in plant-based alternatives—calcium, vitamin B2, vitamin B12, and vitamin D. These four nutrients were chosen because dairy products are either naturally good sources for them (calcium, vitamin B2, and vitamin B12) or are often fortified with them (vitamin D). In the Swiss market, 19% of plant-based milk alternatives (out of 170), 17% of yogurt alternatives (out of 113), and only 3% of cheese alternatives (out of 166) were fortified with calcium. However in the UK, 88% of non-organic milk alternatives (out of 105), 83% of non-organic yogurt alternatives (out of 70), and 54% of non-organic cheese alternatives (out of 66) were fortified with calcium [2]. In a similar market survey conducted in Italy, 38% (out of 330) of the total plant-based milk alternatives were fortified with calcium [44]. For vitamins B2, B12, and D comparisons were similar. While we identified between 8 and 15% of milk and yogurt alternatives fortified with those vitamins, the proportions were much higher, with 20–83%, in the UK survey [2]. Only in fish-alternative products we found fortification with vitamin B12 in Switzerland in 11% of the products, while none were vitamin B12 fortified in the UK [2].

Thus, micronutrient fortification of plant-based dairy and fish-alternative products in Switzerland is relatively low compared to other countries, not only for iodine. However, it has to be noted that the regulations especially for organic products differ between countries, and thus, for our survey we have combined organic and non-organic products altogether, whereas they were strictly separated in the UK survey as fortification of organic products is not allowed in the UK [45–47].

### Limitations and future research

The major limitation of this study is in the lack of laboratory analyses. Our study is based on estimation and factorial calculation and was limited by the use of predominantly manufacturer-supplied information and is not equivalent to independent laboratory analysis. Our values of iodine in unfortified alternatives might not always accurately reflect the actual iodine content in the products as they are dependent on the available information in the food composition table. A second aspect worth mentioning is the ephemerality of the collected data. The results of our survey are applicable to the current market scenario. In the near future, it is expected that the manufacturers will change their product composition, and hence, our study needs to be deemed as a snapshot of the current situation. A final limitation concerns the inclusion of calcium and vitamins B2, B12, and D. Since we have only recorded and reported the availability of products fortified with those nutrients on the marked but have not estimated the potential reduction in intakes by replacing dairy products and fish by plant-based alternatives, we can also not make any judgment about the need of fortification with those nutrients. The main goal of including those nutrients was to have a comparison of fortification levels with that of iodine.

Further research is necessary to understand the impact of the consumption of plant-based alternatives on the iodine adequacy of the population. Future studies involving independent laboratory analyses to evaluate and accurately measure iodine content would be valuable for all types of dairy and fish alternatives. Keeping the list of products on the Swiss Food Composition Database up to date is also important. Also, a study to assess the bioavailability of iodine obtained from plant-based alternatives is required. Lastly, due to a lack of information and difficulty in assessing the use and intake of iodized salt [48], it is required to conduct studies that (representatively) assess the urinary iodine concentration (iodine status) in individuals who consume a significant amount of plant-based alternatives but are not vegan.

To conclude, our cross-sectional study provides a comprehensive overview of the iodine content in dairy and fish products and their fortified and unfortified plant-based alternatives on the Swiss market. Our study not only estimates the iodine intake by modeling dietary scenarios around the Swiss Food Pyramid but it also gives an estimation of iodine intake based on the actual intake of the Swiss adult population. This study has highlighted that only a small proportion of plant-based dairy alternatives are fortified with iodine in the Swiss market. Thus, the part of the population that consumes unfortified plant-based alternatives might be at risk of iodine inadequacy. Food manufacturers and the healthcare sector must come together and increase iodine fortification in plant-based alternatives and also increase awareness among the population about the necessity of iodine from dietary sources.

### Supplementary Information

Below is the link to the electronic supplementary material.

Supplementary file1 (PDF 657 KB)
