# Supplementary material for: Contribution of plant-based dairy and fish alternatives to iodine nutrition in the Swiss diet: a Swiss Market Survey
Source: Eur J Nutr. 2024 Mar 7;63(5):1501–12. doi: 10.1007/s00394-024-03339-5 (PMC11329522; doi:10.1007/s00394-024-03339-5)
Supplement: Supplementary file 1 — Supplementary file1 (PDF 657 KB) [file 394_2024_3339_MOESM1_ESM.pdf]

Contribution of plant-based dairy and fish alternatives to iodine nutrition in the Swiss diet – A Swiss Market Survey

Journal
European Journal of Nutrition

Authors
Zulekha Abbas Khalil[1], Isabelle Herter-Aeberli[1]
ETH Zürich, Institute of Food, Nutrition and Health, Laboratory for Nutrition and Metabolic Epigenetics, 8092 Zürich, Switzerland

Corresponding Author
Dr. Isabelle Herter-Aeberli, ETH Zürich, Institute of Food, Nutrition and Health, Laboratory for Nutrition and Metabolic Epigenetics, 8092 Zürich, Switzerland
isabelle.herter@hest.ethz.ch

Supplementary Material

Supplementary Table S1a: List of plant-based milk alternatives identified in the Swiss market and micronutrient information obtained from the nutrition label on their packaging.

MILK ALTERNATIVES (n = 170)										
**Plant Category**	**Name of the Product**	**Product Description**	**Added Sugar**	**Organic/ Non Organic**	**Net Weight**	**Iodine (per 100 ml or 100 g)**	**Calcium (per 100 ml or 100 g)**	**Vit B2 (per 100 ml or 100 g)**	**Vit B12 (per 100 ml or 100 g)**	**Vit D (per 100 ml or 100 g)**
**Oats (n = 51)**	Alpro Hafer Ohne Zucker	Nature	No	No	1000 ml	-	120 mg	0.21 mg	0.38 µg	0.75 µg
	Alpro This is not Milk, Fettarm, 1.8% Fett, Hafer	1.8% Fat, Low fat	No	No	1000 ml	-	120 mg	-	0.38 µg	0.75 µg
	Alpro This is not Milk, Whole 3.5% Fett, Hafer	3.5% Fat, Full fat	No	No	1000 ml	-	120 mg	-	0.38 µg	0.75 µg
	Alnatura Hafer Drink Barista mit Soja	Barista mit Soja	No	Yes	1000 ml	-	-	-	-	-
	Alnatura Hafer Drink Unsweetened	Nature	No	Yes	1000 ml	-	-	-	-	-

	Alnatura Hafer Mandel Drink	with Almonds	No	Yes	1000 ml	-	-	-	-	-
	Alnavit Bio Glutenfrei Hafer Drink Natur	Gluten-free	No	Yes	1000 ml	-	-	-	-	-
	Beleaf Drink Hafer Nature	Nature	No	No	1000 ml	-	-	-	-	-
	Beleaf Drink Haselnuss und Hafer	with Hazelnut	No	No	1000 ml	-	-	-	-	-
	Beleaf Hafer Barista Drink	Barista	No	No	1000 ml	-	-	-	-	-
	Beleaf Hafer mit Mandel	with Almonds	No	No	1000 ml	-	-	-	-	-
	Biedermann Bio Hafer Drink Barista	Nature	No	Yes	1000 ml	-	-	-	-	-
	Bio Natura Hafer Drink Barista	Barista	No	Yes	1000 ml	-	-	-	-	-
	Dennree Bio Hafer Drink Barista	Barista mit Soja	No	Yes	1000 ml	-	-	-	-	-
	Ecomil Hafer Barista Bio	Nature	No	Yes	1000 ml	-	-	-	-	-
	Hofgut Storzeln Hafer Drink Organic	Nature	No	Yes	1000 ml	-	-	-	-	-
	Isola Bio Hafer Barista Foamable	Barista	No	Yes	1000 ml	-	-	-	-	-
	Isola Bio Hafer Original	Original	No	Yes	1000 ml	-	-	-	-	-
	Jucker Farm Hafer Drink	Nature	No	No	1000 ml	-	-	-	-	-
	Karma Bio Oat Drink Barista	Barista	No	Yes	1000 ml	-	-	-	-	-
	Karma Bio Oat Haferdrink Glutenfrei	Gluten-free	No	Yes	500 ml	-	-	-	-	-
	Karma Bio Oat Haferdrink Mandel	with Almonds	No	Yes	1000 ml	-	-	-	-	-
	Migros V-Love Hafer Drink Barista	Barista	No	Yes	1000 ml	-	-	-	-	-
	Migros V-Love Hafer Drink Calcium	with Calcium	No	No	1000 ml	-	120 mg	-	-	-
	Molkerei Neff Bio Hafer Drink Barista	Barista	No	Yes	1000 ml	-	-	-	-	-
	Natumi Hafer Barista Drink Bio	Barista	No	Yes	1000 ml	-	-	-	-	-
	Nature Active Bio Hafer Barista	Barista	No	Yes	1000 ml	-	-	-	-	-
	Nature Active Bio Hafer Nature	Nature	No	Yes	1000 ml	-	-	-	-	-
	Oatly Bio Hafer Drink	Original	No	Yes	1000 ml	-	-	-	-	-

	Oatly Hafer Drink Calcium	with Calcium	No	No	1000 ml	22.5 µg	120 mg	0.21 mg	0.38 µg	1.1 µg
	Oatly Hafer Drink Classic	Classic	No	No	1000 ml	22.5 µg	120 mg	0.21 mg	0.38 µg	1.1 µg
	Oatly Hafer Drink Deluxe	Deluxe	No	No	1000 ml	22.5 µg	120 mg	0.21 mg	0.38 µg	1.1 µg
	Oatly Hafer Barista Edition	Barista	No	No	1000 ml	22.5 µg	120 mg	0.21 mg	0.38 µg	1.1 µg
	Primavena Hafer Drink Barista Bio	Barista	No	Yes	1000 ml	-	-	-	-	-
	Primavena Hafer Drink Calcio	with Calcium	No	No	1000 ml	-	120 mg	-	-	-
	Primavena Hafer Drink Naturlich Bio	Nature	No	Yes	1000 ml	-	-	-	-	-
	Prix Garantie Hafer Avoine Drink (Oat Milk)	Nature	No	No	1000 ml	-	-	-	-	-
	Provamel Organic Hafer Barista	Barista	Yes	Yes	1000 ml	-	-	-	-	-
	Provamel Organic Hafer Drink	Nature	Yes	Yes	1000 ml	-	-	-	-	-
	Provamel Organic Hafer Drink Ohne Zucker	Nature	No	Yes	1000 ml	-	-	-	-	-
	Provamel Organic Oat Almond Drink	with Almonds	Yes	Yes	1000 ml	-	-	-	-	-
	Soyana Swiss Cereal Bio-Farm Schweizer Hafer Drink Bio	Nature	No	Yes	1000 ml	-	-	-	-	-
	Soyana Swiss Cereal Hafer Barista Drink	Barista	No	Yes	1000 ml	-	-	-	-	-
	Soyana Swiss Cereal Hafer Creamy Drink	Creamy	No	Yes	1000 ml	-	-	-	-	-
	Soyana Swiss Cereal Hafer Drink Bio	Nature	No	Yes	1000 ml	-	-	-	-	-
	Soyana Swiss Cereal Hafer mit Calcium Drink	with Calcium	No	No	1000 ml	-	120 mg	-	-	-
	Spar Natural Organic Oat	Nature	No	Yes	1000 ml	-	-	-	-	-
	Vemondo Bio Oat Drink	Nature	No	Yes	1000 ml	-	-	-	-	-
	Voelkel Hafer Barista Drink	Barista	No	Yes	750 ml	-	-	-	-	-
	Voelkel Hafer Mandel Drink	with Almonds	No	Yes	750 ml	-	-	-	-	-
	Voelkel Hafer Drink	Gluten-free	No	Yes	750 ml	-	-	-	-	-
**Almond**	Alpro Mandel Ohne Zucker	Nature	No	No	1000 ml	-	120 mg	0.21 mg	0.38 µg	0.75 µg

**(n = 19)**										
	Alpro Barista Mandel	Barista	Yes	No	1000 ml	-	120 mg	-	-	-
	Alnatura Mandel Drink Unsweetened	Nature	No	No	1000 ml	-	-	-	-	-
	Beleaf Mandel Drink	Nature	No	No	1000 ml	-	-	-	-	-
	Ecomil Mandelgetrank ohne Zucker mit Kalzium	with Calcium	No	Yes	1000 ml	-	120 mg	-	-	-
	Ecomil Mandelmich ohne Zucker	Nature	No	Yes	1000 ml	-	-	-	-	-
	Isola Bio Almond 0% Sugars	Nature	No	Yes	1000 ml	-	-	-	-	-
	Isola Bio Latte di Mandorla Almond Drink Organic Glutenfrei	Gluten-free	Yes	Yes	1000 ml	-	-	-	-	-
	Isola Organic Almond Drink mit Calcium	with Calcium	No	Yes	1000 ml	-	60 mg	-	-	-
	Karma Bio Mandel Drink	Nature	Yes	Yes	1000 ml	-	-	-	-	-
	Karma Bio Mandeldrink Barista	Barista with Rice	No	Yes	1000 ml	-	-	-	-	-
	M-Budget Mandel Drink	Nature	Yes	No	1000 ml	-	-	-	-	-
	Milbona Bio Organic Mandel Drink	Nature	Yes	Yes	1000 ml	-	-	-	-	-
	Nature Active Bio Mandel Sweetened	Nature	Yes	Yes	1000 ml	-	-	-	-	-
	Nature Active Bio Mandel Unsweetened	Nature	No	Yes	1000 ml	-	-	-	-	-
	Provamel Bio Mandel Barista Drink	Barista	Yes	Yes	1000 ml	-	-	-	-	-
	Provamel Organic Mandel Drink	Nature	Yes	Yes	1000 ml	-	-	-	-	-
	Provamel Organic Mandel Drink Ohne Zucker	Nature	No	Yes	1000 ml	-	-	-	-	-
	Vemondo Almond Drink Unsweetened	Nature	No	Yes	750 ml	-	-	-	-	-
**Soy (n = 23)**	Allos Barista ideal für Kaffee mit Soja Bio	Barista	No	Yes	1000 ml	-	-	-	-	-
	Alnatura Soya Drink Unsweetened	Nature	No	Yes	1000 ml	-	-	-	-	-

	Alpro Barista for professionals Soya	Barista	Yes	No	1000 ml	-	120 mg	0.21 mg	0.38 µg	0.75 µg
	Alpro Barista Soya	Barista	Yes	No	1000 ml	-	120 mg	0.21 mg	0.38 µg	0.75 µg
	Alpro Original Soya Drink with Calcium	with Calcium	Yes	No	1000 ml	-	120 mg	0.21 mg	0.38 µg	0.75 µg
	Alpro Soya Drink Ohne Zucker	Nature	No	No	1000 ml	-	120 mg	0.21 mg	0.38 µg	0.75 µg
	Bio Natura Soja Drink Natur	Drink	Yes	Yes	1000 ml	-	-	-	-	-
	Hofgut Storzeln Soja Drink Organic	Nature	No	Yes	1000 ml	-	-	-	-	-
	Isola Organic Soja Drink ohne Zucker	Nature	No	Yes	1000 ml	-	-	-	-	-
	Isola Organic Soja Barista Drink	Barista	No	Yes	1000 ml	-	-	-	-	-
	Karma Bio Soy Drink	Nature	No	Yes	1000 ml	-	-	-	-	-
	M-Budget Soja Drink Calcium	with Calcium	Yes	No	1000 ml	-	120 mg	0.1 mg	0.2 µg	0.4 µg
	Migros V-Love Soja Drink Nature	Nature	No	Yes	1000 ml	-	-	-	-	-
	Migros V-Love Soja Drink Protein	with Protein	No	Yes	1000 ml	-	-	-	-	-
	Milbona Bio Organic Soja Drink	Nature	Yes	Yes	1000 ml	-	-	-	-	-
	Nature Active Bio Sojadrink Natur	Nature	Yes	Yes	1000 ml	-	-	-	-	-
	Prix Garantie Soja Drink	Nature	Yes	No	1000 ml	-	-	-	-	-
	Provamel Bio Soya Drink Ohne Zucker	Nature	No	Yes	1000 ml	-	-	-	-	-
	Soyana Swiss Soy Drink mit Calcium	with Calcium	Yes	Yes	1000 ml	-	120 mg	-	-	-
	Soyana Swiss Soya Drink Natur	Nature	No	Yes	1000 ml	-	-	-	-	-
	Soyana Swiss Soya Mandeln Drink	with Almonds	Yes	Yes	500 ml	-	-	-	-	-
	Soyana Swiss Soya Original Drink	Original	Yes	Yes	1000 ml	-	-	-	-	-
	Spar Natural Organic Soya	Nature	No	Yes	1000 ml	-	-	-	-	-

**Rice (n = 28)**	Allos Rice Mandel Drink Ungessusst	with Almonds	No	Yes	1000 ml	-	-	-	-	-
	Alnatura Reis Drink Unsweetened	Nature	No	Yes	1000 ml	-	-	-	-	-
	Dennree Bio Reis Drink	Nature	No	Yes	1000 ml	-	-	-	-	-
	Isola Bio Mini Reis Almond Drink	with Almonds	No	Yes	250 ml	-	-	-	-	-
	Isola Bio Reis Haselnuss Drink	with Hazelnut	No	No	1000 ml	-	-	-	-	-
	Isola Organic Brown Rice Drink	Brown Rice	No	Yes	1000 ml	-	-	-	-	-
	Isola Organic Reis Drink	Nature	No	Yes	1000 ml	-	-	-	-	-
	Isola Organic Reis Kokos Drink	with Coconut	No	Yes	1000 ml	-	-	-	-	-
	Karma Bio Rice Drink mit Haselnuss Noisette	with Hazelnut	No	Yes	1000 ml	-	-	-	-	-
	Karma Bio Rice Drink mit Mandel	with Almonds	No	Yes	1000 ml	-	-	-	-	-
	Karma Bio Rice Drink Plain	Nature	No	Yes	1000 ml	-	-	-	-	-
	Lima Reis Haselnuss Mandel Drink	with Hazelnut, Almonds	No	Yes	1000 ml	-	-	-	-	-
	Lima Rice Drink	Nature	No	Yes	1000 ml	-	-	-	-	-
	Migros V-Love Rice Drink Calcium	with Calcium	No	No	1000 ml	-	120 mg	-	-	-
	Migros V-Love Rice Drink Hazelnut	with Hazelnut	No	Yes	1000 ml	-	-	-	-	-
	Migros V-Love Rice Drink Mandel	with Almonds	No	Yes	1000 ml	-	-	-	-	-
	Migros V-Love Rice Drink Nature	Nature	Yes	Yes	500 ml	-	-	-	-	-
	Milbona Bio Organic Rice Drink	Drink	Yes	Yes	1000 ml	-	-	-	-	-
	Nature Active Bio Rice Drink	Drink	Yes	Yes	1000 ml	-	-	-	-	-
	Prix Garantie Reis Drink	Drink	No	No	1000 ml	-	-	-	-	-
	Provamel Organic Reis Drink	Nature	No	Yes	1000 ml	-	-	-	-	-
	Provamel Organic Reis Kokos Drink	with Coconut	No	Yes	1000 ml	-	-	-	-	-

	Soyana Swiss Reis Mandel Drink	with Almonds	Yes	Yes	1000 ml	-	-	-	-	-
	Soyana Swiss Reis VollReis Drink	Whole Rice	No	Yes	1000 ml	-	-	-	-	-
	Soyana Swiss Reis Vollreis Drink mit Calcium	with Calcium	No	Yes	1000 ml	-	120 mg	-	-	-
	Spar Veggie Reis Drink	with Calcium	No	Yes	1000 ml	-	120 mg	-	0.4 µg	0.8 µg
	Spar Veggie Kokos Reis Drink	with Coconut and Calcium	No	No	1000 ml	-	120 mg	-	0.38 µg	0.75 µg
	Vemondo Coconut and Rice Drink	with Coconut	No	Yes	750 ml	-	-	-	-	-
**Coconut (n = 12)**	Alpro Kokonuss Ohne Zucker	Nature	No	No	1000 ml	-	120 mg	-	0.38 µg	0.75 µg
	Alpro Barista Kokonuss	Barista	Yes	No	1000 ml	-	120 mg	-	-	-
	Alnatura Kokos Drink	Nature	No	Yes	1000 ml	-	-	-	-	-
	Alnatura Kokos Drink Gekulht	Chilled	No	No	1000 ml	-	-	-	-	-
	Dr Martins Coco Milk for Drinking Bio	Nature	No	Yes	1000 ml	-	-	-	-	-
	Ecomil Coconut ohne Zucker Drink	Nature	No	Yes	1000 ml	-	-	-	-	-
	Emscha Coco King Bio Kokosmilch Das Original Drink	Original	Yes	Yes	336 ml	-	-	-	-	-
	Emscha Coco King Bio Kokosmilch Fit Balance Drink	Fit Balance	Yes	Yes	336 ml	-	-	-	-	-
	Migros V-Love Kokos Drink Nature	Nature	Yes	Yes	1000 ml	-	-	-	-	-
	Nature Active Bio Kokos	Nature	Yes	Yes	1000 ml	-	-	-	-	-
	Provamel Bio Kokos Barista Drink	Barista	Yes	Yes	1000 ml	-	-	-	-	-
	Provamel Bio Kokos Almond Drink	with Almonds	Yes	Yes	1000 ml	-	-	-	-	-
**Cashew (n = 6)**	Alnatura Cashew Drink Unsweetened	Nature	No	Yes	1000 ml	-	-	-	-	-
	Karma Bio Cashew Drink	Nature	No	Yes	1000 ml	-	-	-	-	-

	Karma Bio Cashew Rice Drink	with Rice	No	Yes	1000 ml	-	-	-	-	-
	Nature Active Bio Cashew	Nature	Yes	Yes	1000 ml	-	-	-	-	-
	Provamel Bio Cashew Drink	Nature	No	Yes	1000 ml	-	-	-	-	-
	Soyatoo Milli! Organic Cashew Drink	Nature	No	Yes	750 ml	-	-	-	-	-
**Pea (n = 7)**	Allos Bio Erbsen Protein Drink	Nature	No	Yes	1000 ml	-	-	-	-	-
	Alnatura Barista Erbsen Drink	Barista	No	Yes	1000 ml	-	-	-	-	-
	Sproud Barista Erbsen Drink	Barista	Yes	No	1000 ml	-	120 mg	0.21 mg	0.38 µg	1.0 µg
	Sproud Unsweetened Powered by Peas	Nature	No	No	1000 ml	-	-	-	-	-
	Vly Barista aus Erbsenprotein	Barista	No	No	1000 ml	-	120 mg	-	-	-
	Vly High Protein aus Erbsenprotein	with Protein	Yes	No	1000 ml	-	120 mg	-	-	-
	Vly Ungesüsst aus Erbsenprotein	Nature	No	No	1000 ml	-	120 mg	-	-	-
**Others (n = 24)**	Alnatura Dinkel Drink	Nature	No	Yes	1000 ml	-	-	-	-	-
	Alnatura Haselnuss Drink Natur	Nature	No	Yes	1000 ml	-	-	-	-	-
	Dennree Bio Dinkel Drink	Nature	No	Yes	1000 ml	-	-	-	-	-
	Ecomil Chufa Erdmandel Drink Natur	Nature	No	Yes	1000 ml	-	-	-	-	-
	Ecomil Hanf Drink Nature Zuckerfrei Bio	Nature	No	Yes	1000 ml	-	-	-	-	-
	Hemi Hanfsamen Drink barista Bio	Barista	Yes	Yes	1000 ml	-	-	-	-	-
	Hemi Hanfsamen Drink Original Bio	Original	Yes	Yes	1000 ml	-	-	-	-	-
	Hemi Hanfsamen Drink Zuckerfrei Bio	Nature	No	Yes	1000 ml	-	-	-	-	-
	Hofgut Storzeln Buchweizen Drink	Nature	No	Yes	1000 ml	-	-	-	-	-
	Isola Organic Hirse Drink	Nature	No	Yes	1000 ml	-	-	-	-	-
	Lupinen Drink Barista - Made with Luve	Barista	Yes	No	1000 ml	-	120 mg	0.21 mg	0.38 µg	0.76 µg

	Lupinen Drink Natur - Made with Luve	Nature	Yes	No	1000 ml	-	-	-	-	-
	Migros V-Love Quinoa Drink Nature	Nature	No	Yes	1000 ml	-	-	-	-	-
	Natumi Buckweizen Natural Drink Bio	Nature	No	Yes	1000 ml	-	-	-	-	-
	Natumi Hirse Natural Drink Bio	Drink	No	Yes	1000 ml	-	-	-	-	-
	Soyana Swiss Cereal Dinkel Drink	Drink	No	Yes	1000 ml	-	-	-	-	-
	Soyana Swiss Cereal Dinkel Haselnuss Drink	with Hazelnut	No	Yes	500 ml	-	-	-	-	-
	Soyana Swiss Cereal Dinkel Plus Calcium Drink	with Calcium	No	Yes	1000 ml	-	120 mg	-	-	-
	Soyana Swiss Cereal Drink Gerste	Nature	No	Yes	1000 ml	-	-	-	-	-
	Soyana Swiss Cereal Hirse Drink	Nature	No	Yes	1000 ml	-	-	-	-	-
	Soyana Swiss Cereal Seven Grains	Nature	No	Yes	1000 ml	-	-	-	-	-
	Allos Ohne Muhh Pflanzendrink 1,5% Fett Bio	with Rice, Coconut and Soy	No	Yes	1000 ml	-	-	-	-	-
	Harvest Moon Milk Alternative Classic 2.1% Fett Bio	with Rice, Coconut and Cashew	No	Yes	1000 ml	-	-	-	-	-
	Harvest Moon Milk Alternative Extra Creamy 3.9% Fett Bio	with Rice, Coconut and Cashew	No	Yes	1000 ml	-	-	-	-	-
**MEDIAN**						**22.5 µg**	**120 mg**	**0.21 mg**	**0.38 µg**	**0.76 µg**
**RANGE**						**22.5**	**60 - 120**	**0.1-0.21**	**0.2-0.4**	**0.4-1.1**
**25th, 75th Percentile[a]**						**22.50, 22.50**	**120, 120**	**0.21, 0.21**	**0.38, 0.38**	**0.75, 1.00**

a: Test for normality using the Shapiro-Wilk test in IBM SPSS [37].

Supplementary S1b: List of plant-based yogurt alternatives identified in the Swiss market and micronutrient information obtained from the nutrition label on their packaging.

YOGURT ALTERNATIVES (n = 113)											
**Plant Category**	**Name of the product**	**Product Type**	**Flavo-ured**	**Added Sugar**	**Organic/ Non-organic**	**Net Weight**	**Iodine (per 100 ml or 100 g)**	**Calcium (per 100 ml or 100 g)**	**Vit B2 (per 100 ml or 100 g)**	**Vit B12 (per 100 ml or 100 g)**	**Vit D (per 100 ml or 100 g)**
**Oats (n =15)**	Beleaf Vegurt Hafer Blueberry	with Blueberry	Yes	Yes	No	150 g	-	120 mg	0.21 mg	0.38 µg	0.75 µg
	Beleaf Vegurt Hafer Schokolade	with Chocolate	Yes	Yes	No	150 g	-	120 mg	0.21 mg	0.38 µg	0.75 µg
	Beleaf Vegurt Oat & Strawberry	with Strawberry	Yes	Yes	No	150 g	-	-	-	-	-
	Biedermann Bio Vegan Hafer Natur	Nature	No	No	Yes	375 g	-	-	-	-	-
	Bio Natura Hafer Crème	Nature	No	No	Yes	400 g	-	-	-	-	-
	Migros V-Love Creamium Apple Cinnamon	with Chickpeas, Apple, Cinnamon	Yes	Yes	No	150 g	-	-	-	-	-
	Migros V-Love Creamium Blueberry	with Chickpeas and Blueberry	Yes	Yes	No	150 g	-	-	-	-	-
	Migros V-Love Creamium Choco	with Chickpeas and Chocolate	Yes	Yes	No	150 g	-	-	-	-	-
	Migros V-Love Vegurt Hafer Schokolade	with Chocolate	Yes	Yes	No	150 g	-	120 mg	0.21 mg	0.38 µg	0.75 µg
	My Vegan Cow Cow vegane Alternative zu Joghurt Vanille	with Vanilla	Yes	Yes	Yes	150 g	-	-	-	-	-
	My Vegan Cow Cow vegane Alternative zu Joghurt Natur	Nature	No	No	Yes	150 g	-	-	-	-	-
	My Vegan Cow Cow vegane Alternative zu Joghurt Kaffee	with Coffee	Yes	Yes	Yes	150 g	-	-	-	-	-
	My Vegan Cow Cow vegane Alternative zu Joghurt Waldfrucht	with Berries	Yes	Yes	Yes	150 g	-	-	-	-	-

	Nestle LC1 Joghurt Pflanzlich Aprikose	with Apricot and Pea protein	Yes	Yes	No	300 g	-	-	-	-	-
	Nestle LC1 Joghurt Pflanzlich Waldbeere	with Forest Berry and Pea Protein	Yes	Yes	No	300 g	-	-	-	-	-
**Almond (n = 12)**	Beleaf Vegurt Almond Nature	Nature	No	No	No	150 g	-	120 mg	0.21 mg	0.38 µg	0.75 µg
	Beleaf Vegurt Almond and Peach	with Peach	Yes	Yes	No	150 g	-	-	-	-	-
	Beleaf Vegurt Almond and Berries	with Berries	Yes	Yes	No	150 g	-	-	-	-	-
	Beleaf Skyr Style Almond & Chocolate	Skyr-style with Chocolate	Yes	Yes	No	150 g	-	-	-	-	-
	Beleaf Skyr Style Almond & Strawberry	Skyr-style with Strawberry	Yes	Yes	No	150 g	-	-	-	-	-
	Bio Natura Mandel Crème	Nature	No	No	Yes	400 g	-	-	-	-	-
	Migros V-Love Vegurt Mandel Himbeere	with Raspberry	Yes	Yes	No	150 g	-	-	-	-	-
	Migros V-Love Vegurt Mandel Mokka	with Mocha	Yes	Yes	No	150 g	-	-	-	-	-
	Migros V-Love Vegurt Mandel Caramel	with Caramel	Yes	Yes	No	150 g	-	-	-	-	-
	Migros V-Love Vegurt Mandel Banane	with Banana	Yes	Yes	No	150 g	-	-	-	-	-
	Alnatura Mandel Natur	Nature	No	No	Yes	400 g	-	-	-	-	-
	Beleaf Vegurt Almond and Mocca	with Mocha	Yes	Yes	No	150 g	-	-	-	-	-
							-				
**Soy (n = 51)**	Alpro Vegan Alternative to Yoghurt Soya-based Mango	with Mango	Yes	No	No	400 g	-	120 mg	0.21 mg	0.38 µg	0.75 µg
	Alnatura Soja Mango Vegan	with Mango	Yes	Yes	Yes	400 g	-	-	-	-	-
	Alnatura Soja Natur	Nature			Yes	400 g	-	-	-	-	-
	Alnatura Soja Vanille Vegan	with Vanilla	Yes	Yes	Yes	400 g	-	-	-	-	-

	Alpro Greek Style Mango Soya Yogurt	Greek-style with Mango	Yes	Yes	No	150 g	-	96 mg	-	0.3 µg	0.6 µg
	Alpro Mehr Frucht Himbeere Apfel	with Raspberry and Apple	Yes	No	No	400 g	-	120 mg	0.21 mg	0.38 µg	0.75 µg
	Alpro Natur Soja	Nature	No	Yes	No	500 g	-	120 mg	-	0.38 µg	0.75 µg
	Alpro Natur Soja Ohne Zucker	Nature	No	No	No	500 g	-	120 mg	-	0.38 µg	0.75 µg
	Alpro Skyr Style	Skyr-style	No	No	No	400 g	-	120 mg	-	0.38 µg	0.75 µg
	Alpro Skyr Style Strawberry	Skyr-style with Strawberry	Yes	Yes	No	400 g	-	120 mg	0.21 mg	0.38 µg	0.75 µg
	Alpro Skyr Style Mango	Skyr-style with Mango	Yes	Yes	No	400 g	-	120 mg	0.21 mg	0.38 µg	0.75 µg
	Alpro Skyr Style Yogurt Vanille	Skyr-style with Vanilla	Yes	Yes	No	400 g	-	120 mg	0.21 mg	0.38 µg	0.75 µg
	Danone Aktivia Vegetable alternative to yogurt	Nature	No	No	No		-	120 mg	-	0.38 µg	0.75 µg
	Karma Fairtrade Bio SoJo Chocolate	with Chocolate	Yes	Yes	Yes	180 g	-	-	-	-	-
	Karma Fairtrade Bio SoJo Mango	with Mango	Yes	Yes	Yes	100 g	-	-	-	-	-
	Karma Fairtrade Bio SoJo Nature	Nature	No	No	Yes	400 g	-	-	-	-	-
	Karma Fairtrade Bio SoJo Plum Cinnamon	with Plum and Cinnamon	Yes	Yes	Yes	180 g	-	-	-	-	-
	Migros V-Love Vegurt Soja Classic	Nature	No	No	Yes	150 g	-	-	-	-	-
	Migros V-Love Vegurt Soja Mango	with Mango	Yes	Yes	Yes	150 g	-	-	-	-	-
	Migros V-Love Vegurt Soja Strawberry	with Strawberry	Yes	Yes	Yes	150 g	-	-	-	-	-
	Milfina Sojagurt Heidelbeere	with Blueberry	Yes	No	No	500 g	-	120 mg	0.21 mg	0.38 µg	0.75 µg
	Milfina Sojagurt Mangue	with Mango	Yes	Yes	No	500 g	-	120 mg	0.21 mg	0.38 µg	0.75 µg
	Milfina Sojagurt Natur	Nature	No	No	No	500 g	-	120 mg	-	0.38 µg	0.75 µg
	Milfina Sojagurt Vanille	with Vanilla	Yes	Yes	No	500 g	-	120 mg	0.21 mg	0.38 µg	0.75 µg
	Provamel Bio Skyr Style Soya Lime Lemon	Skyr-style with Lemon	Yes	Yes	Yes	400 g	-	-	-	-	-

	Provamel Bio Skyr Style Soya Raspberry	Skyr-style with Raspberry	Yes	Yes	Yes	400 g	-	-	-	-	-
	Provamel Organic Bio Skyr Style Soya	Skyr-style Nature	No	No	Yes	400 g	-	-	-	-	-
	Provamel Organic Bio Soya	Nature	No	No	Yes	400 g	-	-	-	-	-
	Provamel Organic Bio Soya Coconut	with Coconut	No	No	Yes	400 g	-	-	-	-	-
	Provamel Organic Bio Soya Red Fruits	with Berries	Yes	Yes	Yes	400 g	-	-	-	-	-
	Provamel Organic Bio Soya Vanilla	with Vanilla	Yes	Yes	Yes	400 g	-	-	-	-	-
	Sojade So Soja! Apricot	with Apricot	Yes	Yes	Yes	400 g	-	-	-	-	-
	Sojade So Soja! Banane	with Banana	Yes	Yes	Yes	400 g	-	-	-	-	-
	Sojade So Soja! Banane Passion Fruit	with Banana and Passion fruit	Yes	No	Yes	400 g	-	-	-	-	-
	Sojade So Soja! Blueberry	with Blueberry	Yes	Yes	Yes	150 g	-	-	-	-	-
	Sojade So Soja! Blueberry Cherry	with Blueberry and Cherries	Yes	No	Yes	400 g	-	-	-	-	-
	Sojade So Soja! Mango	with Mango	Yes	No	Yes	150 g	-	-	-	-	-
	Sojade So Soja! Mango Peach	with Mango and Peach	Yes	Yes	Yes	400 g	-	-	-	-	-
	Sojade So Soja! Natural	Nature	No	No	Yes	400 g	-	-	-	-	-
	Sojade So Soja! Pineapple	with Pineapple	Yes	Yes	Yes	400 g	-	-	-	-	-
	Sojade So Soja! Raspberry and Passion Fruit	with Raspberry and Passion Fruit	Yes	Yes	Yes	150 g	-	-	-	-	-
	Sojade So Soja! Strawberry	with Strawberry	Yes	Yes	Yes	400 g	-	-	-	-	-
	Sojade So Soja! Vanille	with Vanilla	Yes	Yes	Yes	400 g	-	-	-	-	-
	Sojade So Soja! Vegane Alternative zu Quark Vanilla	Quark with Vanilla	Yes	Yes	Yes	400 g	-	-	-	-	-
	Sojade So Soja! Vegane Alternative zu Quark Himbeer	Quark with Raspberry	Yes	Yes	Yes	400 g	-	-	-	-	-

	Sojade So Soja! Vegane Alternative zu Quark Haselnuss	Quark with Hazelnut	No	Yes	Yes	400 g	-	-	-	-	-
	Sojade So Soja! Vegane Alternative zu Quark Nature	Quark Nature	No	No	Yes	400 g	-	-	-	-	-
	Soyana Soyananda Natur Yogurt	Nature	No	No	Yes	250 g	-	-	-	-	-
	Vemondo Soy Yogurt Soya Blueberry	with Blueberry	Yes	Yes	No	501 g	-	-	-	-	-
	Vemondo Soy Yogurt Soya Classic	Nature	No	No	No	500 g	-	-	-	-	-
	Vemondo Soy Yogurt Soya Vanilla	with Vanilla	Yes	Yes	No	502 g	-	-	-	-	-
**Coconut (n = 23)**	Alnatura Kokos Mangue Maracuja	with Mango Maracuja	Yes	Yes	Yes	400 g	-	-	-	-	-
	Alnatura Kokos Natur	Nature	No	No	Yes	400 g	-	-	-	-	-
	Biedermann Bio Vegan Kokos Heidelbeere	with Blueberry	Yes	Yes	Yes	150 g	-	-	-	-	-
	Biedermann Bio Vegan Kokos Mango	with Mango	Yes	Yes	Yes	150 g	-	-	-	-	-
	Biedermann Bio Vegan Kokos Vanille	with Vanilla	Yes	Yes	Yes	150 g	-	-	-	-	-
	Biedermann Bio Vegan Nature	Nature	No	No	Yes	150 g	-	-	-	-	-
	Biedermann Bio Vegan Stracciatella	Stracciatella	Yes	Yes	Yes	150 g	-	-	-	-	-
	Bio Natura Kokos Crème	Nature	No	No	Yes	400 g	-	-	-	-	-
	Gourmand & Végétal Coconut Yogurt	Nature	No	No	No	400 g	-	120 mg	-	-	-
	Gourmand & Végétal Lemon Coconut Yogurt	with Lemon	Yes	Yes	No	120 g	-	120 mg	-	-	-
	Karma Fairtrade Jocos Blueberry	with Blueberry	Yes	Yes	No	150 g	-	-	-	-	-
	Karma Fairtrade Jocos Plain - No added sugar	Nature	No	No	No	400 g	-	-	-	-	-
	Karma Fairtrade Jocos Vanille	with Vanilla	Yes	Yes	No	150 g	-	-	-	-	-
	Migros V-Love Vegurt Coconut Mango Passion Fruit	with Mango and Passion Fruit	Yes	Yes	Yes	150 g	-	-	-	-	-

	Migros V-Love Vegurt Coconut Nature	Nature	No	No	No	150 g	-	-	-	-	-
	Nature Active Bio Kokos Crème	Nature			Yes	400 g	-	-	-	-	-
	Nature Active Bio Kokos Crème Heidelbeere	with Blueberry	Yes	Yes	Yes	400 g	-	-	-	-	-
	Nature Active Bio Kokos Crème Mango	with Mango	Yes	Yes	Yes	400 g	-	-	-	-	-
	Nature Active Bio Kokos Crème Vanille	with Vanilla	Yes	Yes	Yes	400 g	-	-	-	-	-
	Vemondo Coconut Cream Classic	Nature	No	No	No	400 g	-	-	-	-	-
	Vemondo Bio Organic Mangue Auf Basis Von	with Mango	Yes	Yes	Yes	150 g	-	-	-	-	-
	Vemondo Bio Organic Natur Auf Basis Von	Nature	No	No	Yes	150 g	-	-	-	-	-
	Vemondo Bio Organic Pfirsich Auf Basis Von	with Peach	Yes	Yes	Yes	150 g	-	-	-	-	-
							-	-	-	-	-
**Cashew (n = 7)**	Alnatura Cashew Natur	Nature	No	No	Yes	400 g	-	-	-	-	-
	New Roots Bio Coffee	with Coffee	Yes	Yes	Yes	140 g	-	-	-	-	-
	New Roots Bio Strawberry	with Strawberry	Yes	Yes	Yes	140 g	-	-	-	-	-
	New Roots Bio Vanille	with Vanilla	Yes	Yes	Yes	140 g	-	-	-	-	-
	New Roots Vegan Alternative to Yoghurt cashew-based mocha flavour	with Mocha	Yes	Yes	Yes	140 g	-	-	-	-	-
	New Roots Vegan Alternative to Yoghurt Cashew-Based with Chocolate	with Chocolate	Yes	Yes	Yes	140 g	-	-	-	-	-
	New Roots Vegan Alternative to Yogurt Cashew-Based Nature	Nature	No	No	Yes	140 g	-	-	-	-	-
**Others (n = 5)**	Made with Luve aus Lupinen LMGHURT Stracciatella	Stracciatella	Yes	Yes	No	400 g	-	-	-	-	-
	Made with Luve aus Lupinen LMGHURT Vanilla	Vanilla	Yes	Yes	No	400 g	-	-	-	-	-

	Made with Luve aus Lupinen LMGHURT Heidelbeer Cassis	with Blueberry	Yes	Yes	No	400 g	-	-	-	-	-
	Migros V-Love Skyr Style Chickpea Creamy Lemon	Skyr-style with Lemon	Yes	Yes	No	170 g	-	-	-	-	-
	Migros V-Love Skyr Style Chickpea Coconut Blueberry	Skyr-style with Coconut and Blueberry	Yes	Yes	No	170 g	-	-	-	-	-
**MEDIAN**							**NA**	**120 mg**	**0.21 mg**	**0.38 µg**	**0.75 µg**
**RANGE**							**NA**	**96-120**	**0.21**	**0.3-0.38**	**0.6-0.75**
**25th, 75th Percentile [a]**							**NA**	**120, 120**	**0.21, 0.21**	**0.38, 0.38**	**0.60, 0.75**

a: Test for normality using the Shapiro-Wilk test in IBM SPSS [37].

Supplementary Table S1c: List of plant-based milk alternatives identified in the Swiss market and micronutrient information obtained from the nutrition label on their packaging.

CHEESE ALTERNATIVES (n = 166)									
**Plant Category**	**Name of the product**	**Product Type**	**Organic/ Non-Organic**	**Net Weight**	**Iodine (per 100 ml or 100 g)**	**Calcium (per 100 ml or 100 g)**	**Vit B2 (per 100 ml or 100 g)**	**Vit B12 (per 100 ml or 100 g)**	**Vit D (per 100 ml or 100 g)**
**Oats (n = 9)**	Ferretti Cremosa getr. Tomaten pflanzliche Alternative zu Aufstrich	Cheese Spread with Dried Tomatoes and Quinoa	No	200 g	-	-	-	-	-
	Ferretti Cremosa Natur pflanzliche Alternative zu Aufstrich	Cheese Spread with Quinoa	No	200 g	-	-	-	-	-
	Ferretti Donatella pflanzliche Alternative zu Mozzarella	Mozzarella with Quinoa	No	100 g	-	-	-	-	-
	Ferretti Vegiano vegetable alternative to Parmesan	Parmesan with Quinoa	No	200 g	-	-	-	-	-
	Ferretti Vegotta vegetable alternative to ricotta	Ricotta with Quinoa	No	225 g	-	-	-	-	-
	Ferretti Vurata pflanzliche Alternative zu Burrata	Burrata with Quinoa	No	200 g	-	-	-	-	-
	Oatly Hafer Aufstrich Gurke und Knoblauch	Cheese Spread with Cucumber and Garlic	No	150 g	-	-	-	-	-
	Oatly Hafer Aufstrich Natur	Cheese Spread Nature	No	150 g	-	-	-	-	-
	Oatly Hafer Aufstrich Tomate Basilikum	Cheese Spread with Tomato and Basil	No	150 g	-	-	-	-	-
**Almond (n = 26)**	Alnatura Vegane Frischecreme Krauter	Cheese Spread with Herbs	Yes	150 g	-	-	-	-	-
	Alnatura Vegane Frischecreme Natur	Cheese Spread Nature	Yes	150 g	-	-	-	-	-
	Beleaf Frischkase Alternative Mandel Nature	Cheese Spread Nature	No	150 g	-	-	-	-	-
	Green Heart Fresh Cream Natural Organic	Cheese spread Nature	Yes	150 g	-	-	-	-	-
	Green Heart Frischecreme gegrillte Paprika Bio	Cheese spread with Grilled Paprika	Yes	150 g	-	-	-	-	-
	Jay & Joy Jean-Jacques vegane Maroilles-Käse	Maroilles Cheese Nature	Yes	100 g	-	-	-	-	-
	Jay & Joy Joséphine vegane Camembert/Brie	Camembert/Brie Nature	Yes	90 g	-	-	-	-	-

	Jay and Joy Jeanne Le Bleute Vegetal	Blue Cheese Nature	Yes	90 g	-	-	-	-	-
	Jay and Joy Jil	Cheese Block Nature	Yes	120 g	-	-	-	-	-
	Jay and Joy Jil Vegane gereiftem Ziegenkäse	Goat Cheese with Cashew	Yes	90 g	-	-	-	-	-
	Simply V Grill & Pfannen Mediterran	Grill Cheese	No	150 g	-	-	-	-	-
	Simply V Hirtengenuss	White Cheese	No	150 g	-	-	-	-	-
	Simply V Pastagenuss	Shredded Cheese	No	100 g	-	-	-	-	-
	Simply V Reibegenuss	Shredded Cheese	No	200 g	-	-	-	-	-
	Simply V Vegan Streichgenuss Paprika	Cheese Spread with Paprika	No	150 g	-	-	-	-	-
	Simply V Vegan Treat Cremig Milder Streichgenuss	Cheese Spread	No	150 g	-	-	-	-	-
	Simply V Vegan Treat Cremiger Gurke un Knoblauch Streichgenuss	Cheese Spread with Cucumber and Garlic	No	150 g	-	-	-	-	-
	Simply V Vegan Treat Cremiger Krauter Streichgenuss	Cheese Spread with Herbs	No	150 g	-	-	-	-	-
	Simply V Vegane Geniesserscheibe Würzig	Cheese Slices	No	150 g	-	-	-	-	-
	SimplyV Burger Scheiben	Cheese Slices	No	150 g	-	-	-	-	-
	SimplyV Gartenkräuter Veganer Streichgenuss	Cheese Spread with Herbs	No	150 g	-	-	-	-	-
	SimplyV Mild Nutty Natural Gourmet Slices	Cheese Slices	No	150 g	-	-	-	-	-
	Soyana "Melt me" Vegane Mandel-Alternative zu Fondue & Käse-Sauce Bio	Fondue Cheese	Yes	400 g	-	-	-	-	-
	Soyana "Melt me" Vegane Mandel-Alternative zu Weichkäse, Raclette & Topping Bio	Raclette Cheese	Yes	400 g	-	-	-	-	-
	Yolo White Greek	Greek Cheese	No	200 g	-	-	-	-	-
	Zµger Vegan Bio Mozzavella	Mozzarella with Oats	Yes	125g	-	54 mg	-	-	-
**Soy (n = 19)**	Green Heart Fresh Cream Chives	Cheese spread with Chives	Yes	150 g	-	-	-	-	-
	Green Heart Fresh Cream Pomodori Secchi Bio	Pomodor Spread	Yes	150 g	-	-	-	-	-
	Green Heart Vegi Fresh Kräuter Kochcreme Bio	Cheese spread with Herbs	Yes	150 g	-	-	-	-	-

	Green Heart Vegi Fresh Natur Kochcreme Bio	Cheese spread Nature	Yes	150 g	-	-	-	-	-
	Lord of Tofu - Mountain Love Tofu Bio	Pizza melt	Yes	130 g	-	-	-	-	-
	Lord of Tofu - Red Bell Pepper Tofu Bio	Pizza melt with Red Bell Pepper	Yes	130 g	-	-	-	-	-
	Migros V-Love Cottage Cubes Chives	Cottage Cheese	No	180 g	-	-	-	-	-
	Soyana Meerrettich vegane Frischkäse-Alternative Soyananda Bio	Cream Cheese with Horseradish	Yes	140 g	-	-	-	-	-
	Soyana Sonnengetrocknete Tomaten vegane Frischkäse-Alternative Soyananda Bio	Cream Cheese with Sundried Tomatoes	Yes	140 g	-	-	-	-	-
	Soyana Soyananda Fermentierte Bio-Soya Alternative zu Rahmfrischkase	Cream Cheese Nature	Yes	140 g	-	-	-	-	-
	Soyana Soyananda Fermentierte Bio-Soya Vegane Alternative zu Frischkäse Kräuter-Knoblauch	Cream Cheese with Herbs and Garlic	Yes	140 g	-	-	-	-	-
	Soyana Soyananda Fermentierte Bio-Soya Vegane Alternative zu Frischkäse Tomaten	Cream Cheese with Tomato	Yes	140 g	-	-	-	-	-
	Soyana Soyananda Frischkase Pepper	Cream Cheese with Pepper	Yes	140 g	-	-	-	-	-
	Soyananda Alternative zu griechischem Käse Natur	Greek Cheese	Yes	200 g	-	-	-	-	-
	Soyana Vegan Alternative to Greek Cheese & Cashew Pesto Organic	Greek Cheese with Cashew Pesto	Yes	400 g	-	-	-	-	-
	Soyananda Alternative zu griechischem Käse mit Kräutern	Greek Cheese with Herbs	Yes	200 g	-	-	-	-	-
	Soyana Vegane Alternative zu Rahm- und Grillkäse Kräuter	Grill Cream Cheese with Herbs	Yes	200 g	-	-	-	-	-
	Vantastic Foods Grattµgiato Pasta Topping	Pasta Topping	No	60 g	-	-	-	-	-
	Vantastic Foods Grattµgiato Vegane Alternative zu Streukäse auf Sojabasis	Cheese Spread	No	60 g	-	-	-	-	-
**Coconut oil and Starch (n = 52)**	Bedda Bockshornklee Scheiben mit Calcium & Vitamin B12	Fenurgreek Cheese Slices	No	150 g	-	200 mg	-	1.5 µg	-
	Bedda Come on Bert!	Camembert	No	150 g	-	-	-	-	-

	Bedda Granvegano Grated spicy & melt-in-your-mouth	Grated Cheese	No	150 g	-	-	-	-	-
	Bedda Granvegano Vegane Reibekase	Grated Cheese	No	100 g	-	-	-	-	-
	Bedda Hirte in Salzlake Greek Style	Feta Cheese	No	150 g	-	-	-	-	-
	Bedda Kräuter-Hirte in Salzlake Greek Style	Feta Cheese with Herbs	No	150 g	-	-	-	-	-
	Bedda Pfeffer Scheiben mit Calcium & Vitamin B12	Cheese Slices with Pepper	No	150 g	-	200 mg	-	1.5 µg	-
	Bedda Reiberei Classic mit Calcium & Vitamin B12	Grated Cheese Classic	No	150 g	-	200 mg	-	1.5 µg	-
	Bedda Scheiben Bockshornklee	Fenurgreek Cheese Slices (Starch and Coconut oil based)	No	100 g	-	-	-	-	-
	Bedda Scheiben British Style	Cheddar Cheese Slices (Starch and Coconut oil based)	No	150 g	-	-	-	-	-
	Bedda Scheiben Zicke	Goat Cheese Slices (Starch and Coconut oil based)	No	150 g	-	-	-	-	-
	Bedda Scheiben zum schmelzen	Cheese Slices (Starch and Coconut oil based)	No	180 g	-	-	-	-	-
	Bedda Vegarella gerieben fein & mild	Grated Cheese (Starch and Coconut oil based)	No	150 g	-	-	-	-	-
	Bedda Scheiben Classic	Cheese Slices (Starch and Coconut oil based)	No	150 g	-	-	-	-	-
	MEIN VEGGIE TAG The Wonder Burger Schmelzscheiben, vegan	Cheese Slices (Alternate Coconut oil Product)	No	100 g	-	-	-	-	-
	Violife Vegane Alternative zu Emmentaler Geschmack Scheiben	Emmental Cheese slices (Coconut and starch based)	No	140 g	-	-	-	2.5 µg	-
	Violife Vegane Alternative zu Gouda Geschmack Scheiben	Gouda Cheese (Coconut and starch based)	No	140 g	-	-	-	2.5 µg	-
	Vemondo Cream Spread Classic	Cheese Spread	No	150 g	-	-	-	-	-
	Vemondo Cream Spread Herbs	Cheese Spread with Herbs	No	150 g	-	-	-	-	-
	Vemondo Grated Vegan Topping	Grated Topping	No	150 g	-	-	-	-	-
	Vemondo Vegan Greek Style Block	Greek style block	No	150 g	-	-	-	-	-
	Vemondo Vegan Slices British Style	Cheese slices British Style	No	150 g	-	-	-	-	-
	Vemondo Vegan Slices Mediterranean	Cheese Slices Mediterranean	No	150 g	-	-	-	-	-
	Vemondo Vegan Slices Original Flavour	Cheese Slices Original	No	150 g	-	-	-	-	-

	Vemondo Vegan Slices Pepper	Cheese Slices with Pepper	No	150 g	-	-	-	-	-
	Violife mit Cheddar Geschmack	Cheddar Cheese	No	200 g	-	-	-	2.5 µg	-
	Violife mit Cheddar Geschmack Scheiben	Cheddar Slices	No	140 g	-	-	-	2.5 µg	-
	Violife Creamy original flavour	Cream Cheese Original	No	150 g	-	-	-	2.5 µg	-
	Violife Epic Nature Cheddar Flavour Block	Cheddar Block	No	200 g	-	-	-	2.5 µg	-
	Violife Grated	Grated Mozzarella	No	200 g	-	-	-	2.5 µg	-
	Violife Greek White Block	Feta Halloumi	No	200 g	-	-	-	2.5 µg	-
	Violife Mediterranean Block Grill Me!	Grilled Cheese	No	200 g	-	-	-	2.5 µg	-
	Violife Mozzarella Geschmack Gerieben	Shredded Mozzarella	No	200 g	-	-	-	2.5 µg	-
	Violife Original Geschmack Scheiben	Cheese Slices	No	140 g	-	-	-	2.5 µg	-
	Violife Prosociano Wedge	Italian Hard Cheese	No	150 g	-	-	-	2.5 µg	-
	Violife Raucharoma Scheiben	Cheese Slices Smoked	No	140 g	-	-	-	2.5 µg	-
	Violife Scheiben Vegane Alternative zu Mozzarella	Grated Mozzarella	No	200 g	-	-	-	2.5 µg	-
	Wilmersburger Classic	Sliced Bread Topping	No	150 g	-	-	-	-	-
	Wilmersburger Krauter	Sliced Bread Topping	No	150 g	-	-	-	-	-
	Wilmersburger Oregano Thyme Kruiden Herbes	Sliced Bread Topping	No		-	-	-	-	-
	Wilmersburger Pizzaschmelz Bio	Grated Cheese	No	150 g	-	-	-	-	-
	Wilmersburger Scheiben Cheddar-Style	Cheddar Cheese slices	No	150 g	-	-	-	-	-
	Wilmersburger Scheiben Chili	Cheese slices with Chili	No	150 g	-	-	-	-	-
	Wilmersburger Scheiben Classic	Cheese slices Classic	No	150 g	-	-	-	-	-
	Wilmersburger Scheiben Krauter	Cheese slices with Herbs	No	150 g	-	-	-	-	-
	Wilmersburger Scheiben Pepper	Cheese slices with Pepper	No	150 g	-	-	-	-	-
	Wilmersburger Scheiben Pilze	Cheese slices with Mushrooms	No	150 g	-	-	-	-	-
	Wilmersburger Scheiben Tomate-Basilikum	Cheese slices with Tomato and Basil	No	150 g	-	-	-	-	-
	Wilmersburger Scheiben Würzig	Cheese slices Spicy	No	150 g	-	-	-	-	-
	Wilmersburger Stück Classic	Cheese Block Classic	No	300 g	-	-	-	-	-
	Wilmersburger Stück Queen-Style	Cheese Block Queen-style	No	300 g	-	-	-	-	-
	Wilmersburger Stück Würzig	Cheese Block Spicy	No	300 g	-	-	-	-	-
**Rice (n = 7)**	Frescolat MozzaRisella Alternative Blue Bio	Blue Cheese Spread	Yes	150 g	-	-	-	-	-

	Frescolat MozzaRisella Alternative Basil Slices Bio	Cheese Slices with Basil	Yes	80 g	-	-	-	-	-
	Frescolat MozzaRisella SmokeyRisella Bio	Mozzarella Smoked	Yes	200 g	-	-	-	-	-
	Frescolat MozzaRisella Spreadable Classic Bio	Mozzarella Cheese Spread	Yes	80 g	-	-	-	-	-
	Frescolat MozzaRisella Vegane Alternative zu Cheddar-Scheiben Bio	Cheddar Slices	Yes	80 g	-	-	-	-	-
	Frescolat Vegane Alternative zu Mozzarella aus Reis Bio	Mozzarella Classic	Yes	200 g	-	-	-	-	-
	Verys Chicca Vegan Alternative to Cream Cheese	Cream cheese	No	200 g	-	-	-	-	-
**Cashew (n = 32)**	Dr Mannah's Aged Cheese Alternative Chakalaka Bio	Aged Cheese Chakalaka	Yes	100 g	-	-	-	-	-
	Dr Mannah's Aged Cheese Alternative Italian Herbs Bio	Aged Cheese with Italian Herbs	Yes	100 g	-	-	-	-	-
	Dr Mannah's Gereifte Käse Alternative Griechische Kräuter Bio	Aged Cheese with Greek Herbs	Yes	100 g	-	-	-	-	-
	Dr Mannah's Gereifte Käse Alternative Rauch Pfeffer Bio	Aged Cheese with Pepper	Yes	100 g	-	-	-	-	-
	Dr Mannah's Happy White Camembert Alternative Bio	Camembert	Yes	100 g	-	-	-	-	-
	Dr Mannah's Mature Cheese Alternative Herbs de Provence Bio	Mature Cheese with Herbs	Yes	100 g	-	-	-	-	-
	Migros V-Love Plant Based The Softy	Camembert	No	100 g	-	-	-	-	-
	New Roots Black Pepper Organic Spread	Cheese Spread with Black Pepper	Yes	115 g	-	-	-	-	-
	New Roots Garlic and Herbs Organic Spread	Cheese Spread with Garlic and Herbs	Yes	140 g	-	-	-	-	-
	New Roots Greek Style Organic Spread	Greek Cheese Spread	Yes	140 g	-	-	-	-	-
	New Roots Horseradish Organic Spread	Cheese Spread with Horseradish	Yes	140 g	-	-	-	-	-
	New Roots La Cotta Organic Spread	Ricotta	Yes	120 g	-	-	-	-	-
	New Roots La Fondue Organic	Fondue Cheese	Yes	500 g	-	-	-	-	-
	New Roots La Ricotta Olive oil, Thyme and Lavender Organic	Ricotta with Olive Oil, Thyme and Lavender	Yes	120 g	-	-	-	-	-
	New Roots Organic Spread	Cheese Spread	Yes	140 g	-	-	-	-	-

	New Roots Soft White Organic	Soft White Cheese	Yes	120 g	-	-	-	-	-
	New Roots Tomato and Basil Organic Spread	Cheese Spread with Tomato and Basil	Yes	140 g	-	-	-	-	-
	Pa'lais Cucumber & Chive Spread Organic	Cheese spread with Cucumber and Chives	Yes	125 g	-	-	-	-	-
	Pa'lais Garlic & Fine Herb Spread Organic	Cheese spread with Garlic and Herbs	Yes	125 g	-	-	-	-	-
	Pa'lais Nature Aufstrich Organic	Cheese spread Nature	Yes	125 g	-	-	-	-	-
	Simply V Fein Cremige Geniesserscheiben	Cheese Slices	No	150 g	-	-	-	-	-
	Soyana Veganella	Mozzarella	Yes	200 g	-	-	-	-	-
	Soyana Veganella Basilikum	Mozzarella with Basil	Yes	200 g	-	-	-	-	-
	Soyana Veganella Smoked	Mozzarella Smoked	Yes	200 g	-	-	-	-	-
	Soyana Vegane Alternative zu Rahm- und Grillkäse Natur	Grill Cream Cheese Nature	Yes	200 g	-	-	-	-	-
	Cashewrella Plesseblue	Blue Cheese	Yes	170 g	-	-	-	-	-
	Cashewrella Vamembert	Camembert	Yes	155 g	-	-	-	-	-
	Cashewrella Vamembert Feige	Blue Cheese	Yes	155 g	-	-	-	-	-
	Cashewrella Vamembert Pfeffer	Camembert	Yes	155 g	-	-	-	-	-
	Cashewrella Vamembert Truffel	Camembert	Yes	155 g	-	-	-	-	-
	Cashewrella Vamembert Walnuss	Camembert with Walnut	Yes	155 g	-	-	-	-	-
	Cashewrella Vamembert Chili Mango	Camembert with Chili Mango	Yes	155 g	-	-	-	-	-
**Potato (n = 3)**	Veggi Filata Mild	Grated Cheese	Yes	200 g	-	-	-	-	-
	Veggi Filata Naturmild Slices	Cheese Slices Nature	Yes	150 g	-	-	-	-	-
	Pural Vegi Cheezly mit Knoblauch & Kräutern	Cheese block with Garlic and Herbs		190 g	-	-	-	-	-
**Chickpea (n = 5)**	Pangea Food Gondino Vegane Alternative zu Hartkäse gereift Bio	Hard Cheese	Yes	200 g	-	-	-	-	-
	Pangea Food Gondino Vegane Alternative zu Hartkäse Kräuter Bio	Hard Cheese with Herbs	Yes	200 g	-	-	-	-	-
	Pangea Food Gondino Vegane Alternative zu Hartkäse Chili Bio	Hard Cheese with Chilli	Yes	200 g	-	-	-	-	-
	Pangea Food Gondino Alternative zu Hartkäse Tartufo Bio	Hard Cheese Tartufo	Yes	200 g	-	-	-	-	-

	Pangea Food Gondino Alternative zu Hartkäse gerieben Bio	Hard Cheese Grated	Yes	150 g	-	-	-	-	-
**Others (n = 13)**	Migros V-Love The Grilled Herbs	Grilled Cheese	No	180 g	-	-	-	-	-
	Migros V-Love The Melty	Raclette Cheese	No	240 g	-	-	-	-	-
	Migros V-Love The Grated	Grated Cheese	No	200 g	-	-	-	-	-
	Migros V-Love The Classic	Cheese Block	No	180 g	-	-	-	-	-
	Sayve Vegran Natur Bio All Pure	Cheese Block Nature	Yes	400 g	-	-	-	-	-
	Sayve Vegran Walnut Bio	Cheese Block Walnut and Chickpeas	Yes	400 g	-	-	-	-	-
	Sayve Vegran Trüffel Bio	Cheese Block Truffel	Yes	400 g	-	-	-	-	-
	Sayve Vegran Schwarzer Pfeffer Bio	Cheese Block with Pepper	Yes	400 g	-	-	-	-	-
	Sayve Vegran Chili Bio	Cheese Block with Chilli	Yes	400 g	-	-	-	-	-
	Green Heart Rauchige Bohne Aufstrich Bio	Cheese spread Beans	Yes	150 g	-	-	-	-	-
	Simply V Herzhaft Nussige Geniesserscheiben	Cheese Slices Walnut	No	150 g	-	-	-	-	-
	Yolo Cream Nature	Cream Cheese Lupine	No	150 g	-	260 mg	-	1.3 µg	-
	Lupinen Genuss Am Stuck	Cheese Block Lupine	No	180 g	-	-	-	-	-
**MEDIAN**					**NA**	**200 mg**	**NA**	**1.9 µg**	**NA**
**RANGE**					**NA**	**54-260**	**NA**	**1.3-2.5**	**NA**
**25th, 75th Percentile[a]**					**NA**	**54, 200**	**NA**	**1.3, 2.5**	**NA**

a: Test for normality using the Shapiro-Wilk test in IBM SPSS [37].

Supplementary Table S1d: List of plant-based milk alternatives identified in the Swiss market and micronutrient information obtained from the nutrition label on their packaging.

FISH ALTERNATIVES (n = 28)									
**Plant Category**	**Name of the product**	**Product Type**	**Organic/Non-organic**	**Net Weight**	**Iodine (per 100 ml or 100 g)**	**Calcium (per 100 ml or 100 g)**	**Vit B2 (per 100 ml or 100 g)**	**Vit B12 (per 100 ml or 100 g)**	**Vit D (per 100 ml or 100 g)**
**Carrot (n = 3)**	Wild Foods Wood Smoked Ruebli Lax	Salmon	Yes	130 g	-	-	-	-	-
	Wood Smoked Rüebli Lax vegane Alternative zu Lachs mit Alpen-Dill & Pfeffer Bio	Salmon	Yes	130 g	-	-	-	-	-
	Yolo Wild Tatar	Salmon Tatar		175 g	-	-	-	1.25 µg	-
**Pea Protein (n = 3)**	Garden Gourmet Vuna	Tuna		175 g	-	-	-	-	-
	Revo Lachs aus Pflanzen mit Erbsenprotein & Omega-3	Salmon		80 g	-	-	0.4 mg	0.8 µg	1.5 µg
	Veganz Tunno	Tuna		140 g	-	-	-	-	-
**Soy (n = 16)**	Just Veg Vegane Knusperstäbchen	Vegan Fish Sticks		190 g	-	-	-	-	-
	Lord of Tofu Raucherlocken Tofu mit Meeresalgen	Smoked Curls	Yes	180 g	-	-	-	-	-
	Lord of Tofu Meeresliebe Tofu-Krabben	Crabs	Yes	180 g	-	-	-	-	-
	Lord of Tofu Sudsee-Tofu Veganer Hummer-Ersatz	Lobster	Yes	180 g	-	-	-	-	-
	Lord of Tofu Tofu-Atlantik	Atlantik	Yes	150 g	-	-	-	-	-
	Lord of Tofu Tofu-Thuna	Tuna	Yes	110 g	-	-	-	-	-
	Lord of Tofu Tofu-Vegarnelen Creveganettes	Shrimps	Yes	150 g	-	-	-	-	-
	Migros V-Love Plant-Based Sea Style Sticks	Vegan Fish Sticks		300 g	-	-	-	-	-
	PlantTuna mit Chili und Ingwer	Tuna with Chili and Ginger		150 g	-	-	-	-	-
	PlanTuna in Wasser	Tuna		150 g	-	-	-	-	-

	PlanTuna mit Mayo	Tuna with Mayo		150 g	-	-	-	-	-
	PlanTuna mit Mediterranen Krautern	Tuna with Herbs		150 g	-	-	-	-	-
	PlanTuna mit Olivenol	Tuna with Olive oil		150 g	-	-	-	-	-
	PlanTuna mit Zitrone und Pfeffer	Tuna with Lemon and Pepper		150 g	-	-	-	-	-
	Vantastic Food Ocean Fillet	Ocean Fillets Salmon		300 g	-	-	-	-	-
	Vantastic Food Ocean Steak	Veggie Fish Steak		300 g	-	-	-	-	-
**Wheat Protein (n = 2)**	Vemondo Vegan Sticks/Nµggets Sea Style	Vegan Fish Sticks		300 g	-	-	-	-	-
	Vivera Veganes Lachsfilet	Salmon Fillets		200 g	-	-	-	0.71 µg	-
**Starch (n = 4)**	Zeastar Crispy Lemon Shrimpz	Shrimps with Lemon		250 g	-	-	-	-	-
	Zeastar Kalamariz	Calamari		250 g	-	-	-	-	-
	Zeastar No Tuna Sashimi	Tuna Sashimi		230 g	-	-	-	-	-
	Zeastar Zalmon Sashimi	Salmon Sashimi		230 g	-	-	-	-	-
**MEDIAN**					**NA**	**NA**	**0.4 mg**	**0.8 µg**	**1.5 µg**
**RANGE**					**NA**	**NA**	**0.4**	**0.71-1.25**	**1.5**
**25th, 75th Percentile[a]**					**NA**	**NA**	**0.4, 0.4**	**0.71, 1.25**	**1.5, 1.5**

a: Test for normality using the Shapiro-Wilk test in IBM SPSS [37].

Supplementary Table S2a: Nutritional composition of (per 100g) of cow milk from Swiss Food Composition Database [34]**.**

**COW MILK**						
**Food ID**	**Food Name**	**Iodine (µg/ 100g)**	**Calcium (mg/ 100g)**	**Vitamin B2 (mg/ 100g)**	**Vitamin B12 (µg/ 100g)**	**Vitamin D (µg/ 100g)**
1194	Cow milk, average	9.5	120	0.2	0.21	0.1
13399	Milk, half skimmed 1.5 % fat, UHT	10	120	0.2	0.25	0
64	Cow milk, skimmed, UHT	3.1	130	0.16	0.1	0
59	Milk partially skimmed, pasteurized	9.2	120	0.19	0.27	0
61	Milk partially skimmed, UHT	10	120	0.19	0.16	0.1
62	Whole milk, pasteurized	10	120	0.21	0.33	0.1
63	Whole milk, UHT	9.5	120	0.22	0.16	0.1
**MEDIAN**[a]		**9.5**	**120**	**0.2**	**0.21**	**0.1**
**RANGE**		**3.10 - 10**	**120 - 130**	**0.16 - 0.22**	**0.10 - 0.33**	**0.00 - 0.10**
**25th, 75th Percentile**[b]		**9.20, 10.00**	**120, 120**	**0.19, 0.21**	**0.16, 0.27**	**0.00, 0.10**

a: Median values of micronutrients obtained from Swiss Food Composition Database for various cow milk types [34].
b: Test for normality using the Shapiro-Wilk test in IBM SPSS [37].

Supplementary Table S2b: Nutritional composition of (per 100g) of cow milk yogurt from Swiss Food Composition Database.

YOGURT						
**Food ID**	**Food Name**	**Iodine (µg/ 100g)**	**Calcium (mg/ 100g)**	**Vitamin B2 (mg/ 100g)**	**Vitamin B12 (µg/ 100g)**	**Vitamin D (µg/ 100g)**
590	Yogurt flavored, low fat, with sweetener	6	100	0.17	0.4	_
1192	Yoghurt with added sugar (average)	6.1	120	0.15	0.3	0.1
58	Yogurt Bifidus, plain	7.5	150	0.13	0.5	0.2
52	Yogurt, Natural	16	140	0.18	0.3	0.1
53	Yogurt, Strawberry	5.5	110	0.15	0.27	0.1
54	Yogurt, Chocolate	6	130	0.12	0.3	0.1
55	Yogurt, Hazelnut	6.5	110	0.13	0.4	0.1
56	Yogurt, Vanilla	6.5	130	0.14	0.4	0.1
57	Yogurt, Mocca	6	130	0.22	0.15	0.1
99	Yogurt, Mocca organic	6	120	0.16	0.3	0.1
587	Yogurt, low fat	9.5	160	0.26	0.44	0.1
588	Yogurt with fruit, low fat with sweetener	6	110	0.17	0.4	_
571	Quark, natural, semi-fat	21	110	0.34	0.37	0.1
**MEDIAN[a]**		**6.1**	**120**	**0.16**	**0.37**	**0.1**
**RANGE**		**5.5 - 21.00**	**100 - 160**	**0.12 - 0.34**	**0.15 - 0.50**	**0.00 - 0.20**
**25th, 75th Percentile[b]**		**6.00, 8.50**	**110, 135**	**0.13, 0.2**	**0.30, 0.40**	**0.10, 0.10**

a: Median values of micronutrients obtained from Swiss Food Composition Database for various cow milk yogurt types [34].
b: Test for normality using the Shapiro-Wilk test in IBM SPSS [37].

Supplementary Table S2c: Nutritional composition of (per 100g) of cow milk cheese from Swiss Food Composition Database.

CHEESE						
**Food ID**	**Food Name**	**Iodine (µg/ 100g)**	**Calcium (mg/ 100g)**	**Vitamin B2 (mg/ 100g)**	**Vitamin B12 (µg/ 100g)**	**Vitamin D (µg/ 100g)**
554	Appenzeller, at least 15% fidm	35	1090	0.5	1.3	0.3
553	Appenzeller, at least 45% fidm	8.4	740	0.36	1.06	0.5
1211	Bernese alp cheese	n.d.	920	n.d.	n.d.	n.d.
1212	Bernese cheese for slicing	n.d.	n.d.	0.56	0.83	0.8
13395	Blanc battu, plain, low fat	10	130	0.32	0.36	0.1
1191	Blue cheese (average)	40	610	0.58	0.72	0.5
613	Brie, at least 45% fidm	8.3	420	0.25	1.8	0.5
616	Brie, at least 50% fidm	20	500	0.34	1.31	0.7

622	Camembert, at least 25% fidm	20	530	0.5	1.79	0.5
614	Camembert, at least 45% fidm	20	370	0.23	1.8	0.5
619	Camembert, at least 50% fidm	6.8	350	0.71	1.94	0.5
1196	Cheese grated	40	1190	0.34	1.91	0.7
505	Cottage cheese	20	69	0.23	0.52	0.1
567	Cream cheese, min. 60% fidm	8	110	0.26	0.6	tr.
555	Emmentaler cheese, at least 45% fidm	20	1030	0.3	1.67	0.5
539	Feta, cow milk	tr.	480	0.33	0.88	0.3
483	Gorgonzola	40	610	0.36	0.9	0.6
530	Greyerzer, at least 45% fidm	11	900	0.37	1.45	0.7
1193	Hard and semi hard cheese, full fat (average)	16	910	0.34	1.5	0.6
531	Limburger (Münster) cheese	20	230	0.46	2.18	0.4
82	Mozzarella	4.8	340	0.27	1.4	0.3
482	Parmesan cheese	40	1340	0.29	1.75	0.8
558	Processed cheese, 15% fidm, slice	35	600	0.32	1.62	0.2
560	Processed cheese, 15% fidm, spreadable	35	600	0.38	2	0.2
559	Processed cheese, 45% fidm, slice	35	600	0.23	1.17	0.7
561	Processed cheese, 45% fidm, spreadable	35	500	0.38	2	0.5
563	Processed cheese, 50% fidm, slice	35	550	0.37	2	0.9
565	Processed cheese, 50% fidm, spreadable	35	400	0.37	2	0.6
532	Raclette cheese	11	670	0.29	1.55	0.3
533	Reblochon cheese	tr.	390	0.36	1.5	0.2
484	Roquefort cheese	40	600	0.81	0.54	0.4
552	Sbrinz	40	1030	0.39	2.07	0.6
507	Schabziger (Swiss green cheese)	tr.	860	0.28	tr.	tr.
1197	Soft cheese (average without fat reduced products)	17	440	0.43	1.45	0.4
13391	Soft cheese, 45 to 54.9% fidm	7.1	360	0.36	1.4	0.5
631	Soft cheese, 60% fidm	20	270	0.38	1.5	0.7
13392	Soft cheese, at least 55% fidm	11	420	0.64	1.47	0.6
485	Soft cheese, Mascarpone	29	68	0.26	0.33	0.5
570	St. Paulin cheese	35	700	0.32	2	0.5
495	Tête de Moine	28	700	0.27	1.6	0.7
556	Tilsiter, pasteurized milk, at least 45% fidm	7.3	840	0.27	1.64	0.5
557	Tilsiter, raw milk, at least 45% fidm	7.9	900	0.38	1.33	0.6

615	Tomme cheese	tr.	290	0.4	1.8	0.2
536	Vacherin Fribourgeois	27	640	0.33	1.62	0.4
534	Vacherin Mont d'or	20	430	0.26	2.06	0.5
506	Ziger white	tr.	170	tr.	tr.	tr.
**MEDIAN[a]**		**20**	**550**	**0.35**	**1.5**	**0.5**
**RANGE**		**0.00 - 40**	**68 - 1340**	**0.00 - 0.81**	**0.00 - 2.18**	**0.00 - 0.9**
**25th, 75th Percentile[b]**		**10.50, 35.00**	**365, 790**	**0.28, 0.38**	**1.08, 1.8**	**0.32, 0.60**

a: Median values of micronutrients obtained from Swiss Food Composition Database for various cow milk cheese types [34].
b: Test for normality using the Shapiro-Wilk test in IBM SPSS [37].

Supplementary Table S2d: Nutritional composition of (per 100g) of fish from Swiss Food Composition Database.

**FISH**						
**Food ID**	**Food Name**	**Iodine (µg/ 100g)**	**Calcium (mg/ 100g)**	**Vitamin B2 (mg/ 100g)**	**Vitamin B12 (µg/ 100g)**	**Vitamin D (µg/ 100g)**
1023	Black salsify, steamed (without addition of salt)	2.6	56	0.04	0	0
1098	Fish (average), raw	45	23	0.13	5.31	6.3
13345	Fish (average), filet, steamed (without addition of fat and salt)	50	29	0.11	5.31	6.5
760	Fish, hake, raw	120	25	0.08	2.1	
416	Fish, plaice, raw	53	61	0.22	1.5	3
754	Fish, pollock, raw	85	8	0.05	1.3	0.8
757	Fish, sardine, raw	32	85	0.25	6	11
755	Fish, sole, raw	25	29	0.05	0.8	8
756	Fish, tuna, raw	50	28	0.12	6.8	4.2
1099	Shellfish (average), raw	170	30	0.05	0.45	0
211	Whitefish, raw	1.5	34	0.07	1	22.1
13354	Salmon, cultured, filet, steamed (without addition of fat and salt)	49	15	0.12	6.9	7.3
194	Salmon, cultured, raw	44	12	0.14	6.9	8.3
193	Salmon, smoked	44	12	0.14	6.9	8.3
210	Salmon, wild, raw	53	8	0.15	6.9	8.4
13350	Cod, filet, steamed (without addition of fat and salt)	180	16	0.04	1.4	2
285	Cod, raw	160	13	0.05	1.4	1.8
765	Eurasian perch, raw	4	80	0.1	1	0.4

773	Fish fingers (sticks), breaded and pre deep fried	89	10	0.04	0.88	0.9
1169	Fish fingers/sticks (breaded and pre deep fried), oven baked (without addition of fat)	100	12	0.04	0.9	0.8
13351	Flounder, filet, steamed (without addition of fat and Salt)	7.1	60	0.18	1	0.2
481	Flounder, raw	6.3	48	0.21	1	0.2
753	Halibut, raw	1	15	0.05	0.96	9
416	Pike, raw	8	32	0.06	2	2
289	Trout, raw	14	14	0.06	5	n.d.
13349	Trout, whole, boiled (without addition of fat and salt)	15	18	0.05	5	n.d.
418	Tuna in oil, drained	43	10	0.1	5	2.4
417	Tuna in water, drained	tr.	9	0.07	3	2.2
419	Fish product, anchovy in oil, drained	30	290	0.25	20.9	1.7
413	Fish product, sardine in oil, drained	26	400	0.25	12	6
1168	Fish fingers/sticks (breaded and pre deep fried), fried in HOLL rapeseed oil	97	11	0.04	0.86	0.8
**MEDIAN[a]**		**44**	**23**	**0.08**	**2**	**2.3**
**RANGE**		**0.00 – 180**	**8 – 400**	**0.04 – 0.25**	**0.00 – 20.9**	**0.00 – 22.1**
**25th, 75th Percentile[b]**		**14.00, 85.00**	**12.00, 48.00**	**0.05, 0.14**	**1.00, 6.00**	**0.8, 7.47**

a: Median values of micronutrients obtained from Swiss Food Composition Database for various cow milk types [34].
b: Test for normality using the Shapiro-Wilk test in IBM SPSS [37].

Supplementary Table S3: Median iodine concentration of unfortified plant-based alternatives, calculated factorially based on characterising plant ingredient.

**Category**	**Plant Type**	**Median iodine concentration in each plant type**	**Median iodine of each category of product[a]**	**25th, 75th Percentile[b]**
**MILK**	Oat	0.066	0.21175	0.074, 0.38
	Almond	0.084		
	Soy	0.521		
	Rice	0.329		
	Coconut	0.096		

	Cashew	0.3275		
	Pea	0.0705		
	Others	0.4		
**YOGURT**	Oat	0.05	0.36205	0.04, 0.71
	Almond	0.018		
	Soy	0.6741		
	Coconut	0.76		
	Cashew	0.7		
	Others	0.0473		
**CHEESE**	Oat	0.04	0.10545	0.047, 2.04
	Almond	0.0215		
	Soy	3.84		
	Coconut oil and Starch	0.1215		
	Rice	0.3952		
	Cashew	2.6		
	Potato	-		
	Chickpea	0.0894		
	Others	0.068		
**FISH**	Carrot	1.472	0.5715	0.27, 2.39
	Pea	0.234		
	Soy	3.3075		
	Wheat	0.5715		
	Starch	0.316		

a: Concentration of iodine from unfortified products calculated factorially. The Swiss Food Composition Database was used to derive the iodine content in 100 g of the characterizing ingredient [34].
b: Test for normality using the Shapiro-Wilk test in IBM SPSS [37].

Supplementary Table S4: Iodine content of characterising ingredients used in unfortified plant-based alternative products from the Swiss Food Composition Database [34].

**Food ID**	**Plant-based product**	**Ingredient**	**Iodine content (µg/100 g)[a]**
273	Almond-based products	Almond whole	0.2
275	Cashew-based products	Cashew nuts, kernel only, plain	5
284	Coconut-based products	Coconut, flesh only, fresh	1.2
13458	Coconut-based products	Coconut milk	1
198	Oat-based products	Oat Flakes	0.5
427	Rice-based products	Rice polished raw	1.9
442	Soya-based products	Soybean, dried	6.3
13389	Pea-based products	Pea, mature seed, split, dried	1.3
270	Hazelnut-based products	Hazelnut	6.5
1042	Dinkel-based products	Spelled flour, whole grain	0.9
421	Buckwheat-based products	Buckwheat, shelled grain	0.5
422	Millet-based products	Millet, shelled grain	2.5
13394	Quinoa-based products	Quinoa, raw	1.6
739	Barley-based products	Barley flakes	1
439	Chickpea-based products	Chickpea, dried	0.6
13438	Tofu-based products	Tofu, soft, plain	0.7
13437	Tofu-based products	Tofu, firm, nature	6
601	Coconut oil-based products	Coconut fat	0
497	Potato starch-based products	Potato starch	1
426	Maize starch-based products	Maize starch	2.5
897	Wheat starch-based products	Wheat starch	0
271	Walnuts-based products	Walnuts	5
13388	Beans-based products	Beans cooked	0.4
355	Carrot-based products	Carrot, raw	1.6
13466	Seaweed-based products	Seaweed, Nori, dried	5100
734	Wheat-based products	Wheat, soft, whole grain	6.7
750	Breadcrumbs-based products	Breadcrumbs	2.8

a: The Swiss Food Composition Database was used to derive the iodine content in 100 g of the characterizing ingredient [34].
